# Elevated Autoantibodies in Subacute Human Spinal Cord Injury Are Naturally Occurring Antibodies

**DOI:** 10.3389/fimmu.2018.02365

**Published:** 2018-10-11

**Authors:** Angel Arevalo-Martin, Lukas Grassner, Daniel Garcia-Ovejero, Beatriz Paniagua-Torija, Gemma Barroso-Garcia, Alba G. Arandilla, Orpheus Mach, Angela Turrero, Eduardo Vargas, Monica Alcobendas, Carmen Rosell, Maria A. Alcaraz, Silvia Ceruelo, Rosa Casado, Francisco Talavera, Ramiro Palazón, Nuria Sanchez-Blanco, Doris Maier, Ana Esclarin, Eduardo Molina-Holgado

**Affiliations:** ^1^Laboratory of Neuroinflammation, Hospital Nacional de Paraplejicos, SESCAM, Toledo, Spain; ^2^Center for Spinal Cord Injuries, Trauma Center, Murnau, Germany; ^3^Department of Neurosurgery, Trauma Center, Murnau, Germany; ^4^Spinal Cord Injury and Tissue Regeneration Center Salzburg, Institute of Molecular Regenerative Medicine, Paracelsus Medical University, Salzburg, Austria; ^5^Proteomics Core Facility, Hospital Nacional de Paraplejicos, SESCAM, Toledo, Spain; ^6^Department of Physical Rehabilitation, Hospital Nacional de Paraplejicos, SESCAM, Toledo, Spain; ^7^Department of Occupational Health, Hospital Nacional de Paraplejicos, SESCAM, Toledo, Spain; ^8^Intensive Care Unit, Hospital Nacional de Paraplejicos, SESCAM, Toledo, Spain

**Keywords:** spinal cord injury, neurotrauma, natural autoantibodies, proteomics, autoimmunity

## Abstract

Spinal cord injury (SCI) results in long-term neurological and systemic consequences, including antibody-mediated autoimmunity, which has been related to impaired functional recovery. Here we show that autoantibodies that increase at the subacute phase of human SCI, 1 month after lesion, are already present in healthy subjects and directed against non-native proteins rarely present in the normal spinal cord. The increase of these autoantibodies is a fast phenomenon–their levels are already elevated before 5 days after lesion–characteristic of secondary immune responses, further supporting their origin as natural antibodies. By proteomics studies we have identified that the increased autoantibodies are directed against 16 different nervous system and systemic self-antigens related to changes known to occur after SCI, including alterations in neural cell cytoskeleton, metabolism and bone remodeling. Overall, in the context of previous studies, our results offer an explanation to why autoimmunity develops after SCI and identify novel targets involved in SCI pathology that warrant further investigation.

## Introduction

Spinal cord injury (SCI) results in neurological and systemic alterations that dramatically interfere with patient quality of life. Several factors contributing to SCI pathology have been proposed in an attempt to identify novel targets for therapeutic intervention. One of these are autoantibodies (AAb) against central nervous system antigens, which are elevated in SCI patients and are pathogenic in rodents (1–5). In mice, SCI induces B cells to proliferate and differentiate into immunoglobulin (Ig) G secreting B cells, which results in increased levels of IgG in peripheral blood (3). Injecting these circulating IgG into the spinal cord of intact animals induces necrosis, inflammation and motor impairment (5). Accordingly, mice genetically devoid of B cells, which do not produce antibodies, develop smaller lesions and recover better from SCI. Although the body of evidence suggest that suppressing antibody production could be an effective therapeutic strategy to promote recovery, paradoxically, SCI patients also develop a well-defined immune depression syndrome that increases their susceptibility to infections, the major cause of morbidity and mortality after SCI (6, 7). Like in humans, mice show impaired immune responses after SCI –including decreased antibody production– that increase their susceptibility to infections (8–10). Consequently, therapies directed to interfere with antibody production and/or their effector mechanisms should not be based on a general immunosuppression, but specifically directed toward those responses related to SCI pathology. So far, significant increased levels of AAb have been described against myelin basic protein (MBP), ganglioside GM1 (GM1), myelin galactocerebroside (GalC), glial fibrillary acidic protein (GFAP) and collapsing response mediator protein-2 (CRMP-2) in humans after SCI (1, 2, 4, 11–13). Also, by incubating patient blood sera with a phage display of a human spinal cord cDNA expression library, a set of five AAbs against 26S proteasome non-ATPase regulatory subunit 4 (PSMD4), glyceraldehyde-3-phosphate dehydrogenase (G3P), myeloma-overexpressed gene protein (MYEOV2), protein S100-B (S100B) and adipocyte enhancer-binding protein 1 (AEBP1) has been proposed to be enriched in patients, although individually none of these AAbs reached statistical significance (14). However, the complete repertoire of AAb specificities induced after SCI remains unknown because most studies have been done by selecting the antigenic determinants to be tested “*a priori*,” not by unguided discovery methods.

Why immune depression and autoimmunity co-occur after SCI remains unsolved. A proposed explanation is that autoimmunity is inversely related to the magnitude of immune depression (6). Immune depression after SCI is more pronounced after cervical and high thoracic lesions (injuries above the 5th thoracic spinal segment) due to the dysregulation of the sympathetic nervous system below the lesion site (10, 15). Accordingly, it has been reported that high thoracic lesions, but not low thoracic, inhibit the production of antibodies after immunization with exogenous antigens in mice (8, 15). However, cervical injuries increase AAb levels against spinal cord self-antigens (16). These opposed results between the responses of antibodies against exogenous and self-antigens may be explained by the observations in rodent experimental models showing that (i) SCI only impairs the generation of new antibody responses but preserves already existing immunity (17) and that (ii) AAbs detected after SCI may exist before lesion (18). Therefore, AAbs observed after SCI could be the result of secondary immune responses of previously existing immunity; i.e., these AAbs could be naturally occurring AAbs. These are normally present in healthy subjects as IgMs and IgGs (19–24) and have been suggested to be either involved in homeostatic and healing processes by recognizing proteins with modifications induced by cell stress or damage (25–27) as well as being the origin of pathological AAbs (25, 28–30).

By using a hypothesis-free methodology we show that AAbs against damaged spinal cord are already detectable in the sera of normal subjects and that AAbs against 16 different targets are significantly increased in the subacute phase after SCI. Among these AAb targets, 13 are reported for the first time to be increased after SCI and unveil a portrait of cellular and molecular alterations with therapeutic interest for SCI.

## Materials and methods

### Patients and healthy control subjects

Fifty-two patients with traumatic SCI and ten patients with traumatic central cord syndrome were recruited at Hospital Nacional de Paraplejicos (Toledo, Spain)—a national center specialized in spinal cord injury—and at Trauma Centre Murnau (Bavaria, Germany)—a cross-regional trauma center with a specialized spinal cord injury department. All patients included in this study were examined at a median time of 31 ± 1 days after injury, and a subgroup of eleven patients from the fifty-two SCI individuals were additionally examined at a mean time of 2 ± 0.4 days after injury (range 0–5 days). All patients fulfilled the inclusion and exclusion criteria and gave their informed consent to participate. Inclusion criteria were:

- Males and females
- At least 18 years old
- Any neurological level of injury, but cauda equine syndrome
- Complete and incomplete lesions
- If patient was treated with glucocorticoids, it should have passed at least 7 days after ending the treatment

Exclusion criteria were:

- Diagnosed autoimmune disorder
- Diagnosed tumor
- Diagnosed neurodegenerative disease

Sensorimotor function of patients was evaluated following the International Standards for Neurological Classification of Spinal Cord Injury scale (ISNCSCI). All evaluations were performed by trained personnel with experience certified by the centralized ISNCSCI training course at Heidelberg University Hospital. Lesion level and severity of patients is summarized in Table 1.

**Table 1:** Lesion level and severity of patients.

**NLI^a^**	**AIS A (#)**	**AIS B (#)**	**AIS C (#)**	**AIS D (#)**
Cervical	14	1	6	1
Thoracic	23	2	1	0
Lumbar	2	1	1	0

a *NLI, neurological level of injury*.

Age and gender-matched voluntary healthy individuals were recruited by the Department of Occupational Health at Hospital Nacional de Paraplejicos (Table 2).

**Table 2:** Demographical characteristics of patients and control subjects.

	***n***	**Age, years (mean ±sem)**
SCI^a^ total	52	40.6 ± 2.2
Men	46	39.1 ± 2.3
Women	6	52.3 ± 8.1
CTL^b^ total	16	45 ± 2.8
Men	13	45.0 ± 3.2
Women	3	47.7 ± 6.7

a *SCI, spinal cord injury patients*.

b *CTL, control healthy subjects*.

The study protocol and the informed consent sheet were evaluated and approved by the Ethics Committee of the Toledo Health Care Area and by the Ethics Committee of the Bavarian Medical Board. The study follows and adheres to the World Medical Association Declaration of Helsinki and is registered at the public database Clinicaltrial.gov (registration number NCT02493543).

### Blood sera

Peripheral blood was collected in anti-coagulant free tubes by venepuncture at the medial cubital vein. Blood clot was allowed to form by maintaining the tubes for 45 min at room temperature (RT) and 1 h at 4°C. Blood was centrifuged at 1,500 g for 20 min at 4°C and serum was collected, aliquoted and stored at −80°C until used.

Whenever a blood sample was taken, a standard hematological analysis including red and white cells count and related parameters determination was performed by the clinical laboratories at Hospital Nacional de Paraplejicos (Toledo, Spain) or at Murnau Trauma Centre (Murnau, Germany).

### Animals and experimental spinal cord injury (SCI)

Young adult male Wistar rats (295–315 g, 12 weeks of age) were obtained from Harlan-Interfauna Iberica (Barcelona, Spain) and were maintained in our animal facilities on a 12:12-h light:dark cycle, receiving food and water *ad libitum*. All experimental procedures were approved by our institutional animal use and care committee. Rats were handled in accordance with the guidelines published by Spain and the European Union (RD1201/2005, 2010/63/EU).

SCI at T8 vertebral level was induced as previously described (31). Briefly, we anesthetized rats with an intraperitoneal injection of sodium pentobarbital (45 mg/Kg, Normon Veterinary Division, Madrid, Spain) and Xylacine (10 mg/Kg, Calier, Barcelona, Spain). After confirming the absence of reflexes, we injected atropine (50 μg/Kg, Brown Medical, Barcelona, Spain) and we applied artificial tears to prevent corneal abrasion and infection. We performed a laminectomy of T8 vertebra and we stabilized the vertebral column by clamping spiny processes of T7 and T9 vertebras. Spinal cord contusion/short compression was performed with the “Infinite Horizon™” device (Precision Systems and Instrumentation, Lexington, KY, USA), applying a force of 200 Kdyn and a compression time of 5 seconds over the exposed cord. Force and displacement curves generated by the impactor were checked to confirm that injuries were done with similar values and profiles without artifacts indicative of an erroneous/abnormal lesion.

Post-operative care included analgesia (Buprenorphine), prophylactic antibiotic treatment (Enrofloxacine) and hydration (saline serum) during the first week after injury. Bladder was manually voided until animals were sacrificed.

**Figure 1:**
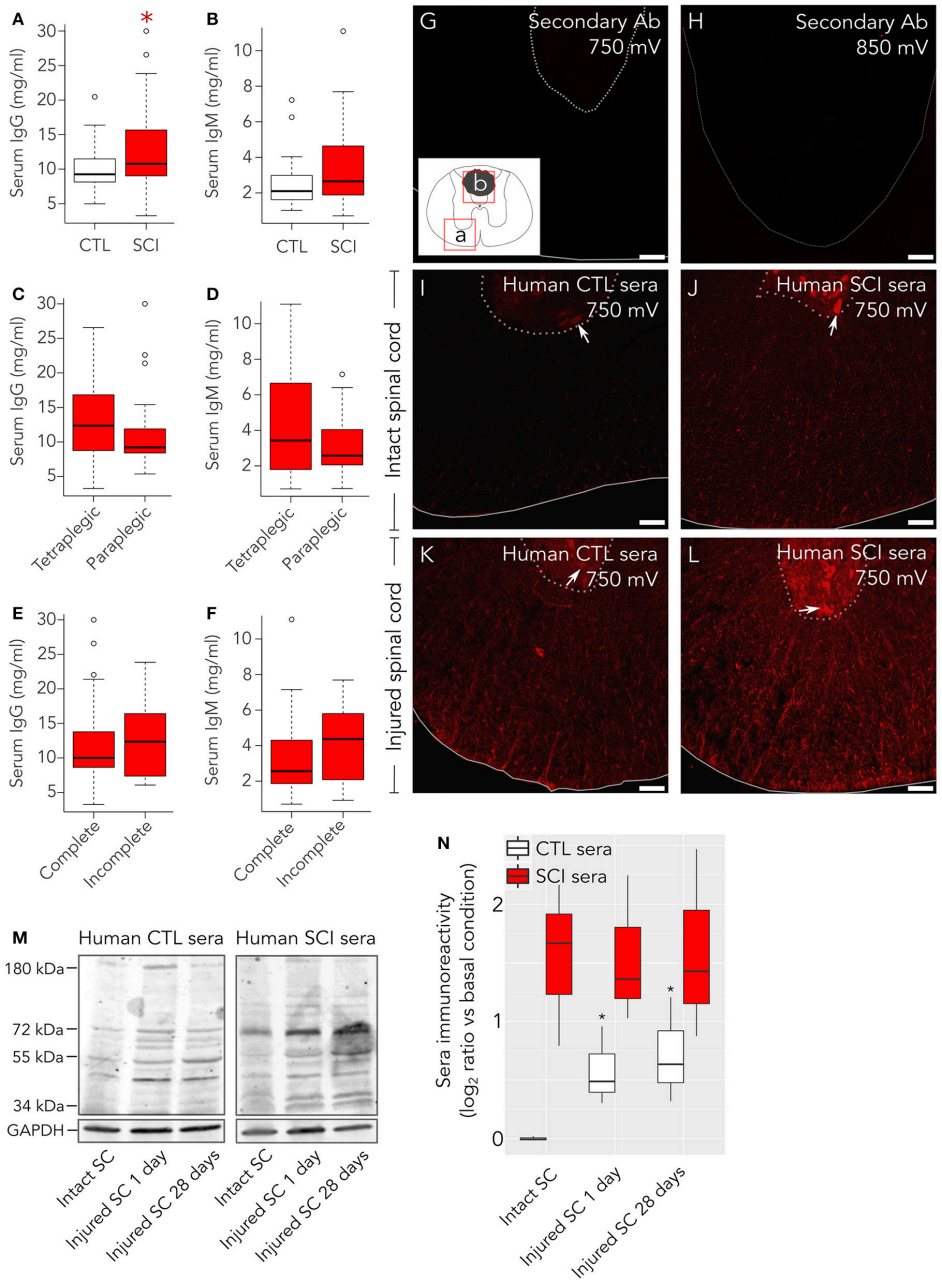
Healthy control subjects present autoantibodies preferentially directed against damaged spinal cord. As measured by ELISA, **(A)** serum IgG concentration is increased in patients compared to control healthy individuals, while **(B)** serum IgM levels are not statistically different between both groups. Neurological level of lesion **(C,D)** and severity (AIS **A** complete vs. sensory incomplete; **E,F**) do not significantly affect the levels of IgGs nor IgMs in patients. **(G–L)** Immunohistochemistry was performed to detect binding of serum IgGs to intact and injured rat spinal cord. All experiments were performed in parallel, to avoid potential batch effects. Confocal microscopy images (single confocal plane) were acquired by setting up photomultiplier voltage (sensitivity) to values far beyond those where unspecific background may arise. **(G)** With confocal microscope photomultiplier at 750 mV no signal is observed on injured spinal cord when only secondary anti-human IgG is added. **(H)** Even at higher intensities, 850 mV, in the lesion border, where autofluorescence is more intense, no signal is detected in these experiments. Rectangles **(a,b)** in the insert of panel **(G)** indicate the spinal cord regions shown in panels **(G,I–L) (a)** or in panel H **(b)**. **(I)** Serum IgGs form control individuals weakly react with intact rat spinal cord, preferentially binding to motoneurons in the ventral horn (arrow), but strongly react with glial and neuronal cellular profiles in injured rat spinal cord **(K)**. Serum IgGs from SCI individuals react with intact tissue **(J)** and with injured spinal cord **(L)**. **(M)** Representative Western blots showing reactivity of serum IgGs from control individuals (CTL, left panel) or spinal cord injury patients (SCI, right panel) over protein extracts from intact rat spinal cord (SC), injured rat SC at 1 days after lesion or injured rat SC at 28 days after injury. **(N)** Quantification of overall IgG immunoreactivity in Western blots show that CTL serum IgGs (*n* = 3) present a significant higher immunoreactivity against proteins isolated from injured rat spinal cord than against proteins isolated from intact rat spinal cord. On the other hand, SCI sera IgGs (*n* = 3) show higher immunoreactivity than control serum IgGs at any condition (2-way ANOVA *p* = 0.006). Basal condition is defined as the mean value of CTL serum IgG immunoreactivity against proteins isolated from intact rat spinal cord. Gray line in **(G,I–L)** marks spinal cord border, and pointed-line delimits the ventral horn. Gray line in **H** depicts lesion border. Arrows in **C–F** point to ventral horn motoneurons. ^*^p < 0.05. Scale bar, 75 μm.

**Figure 2:**
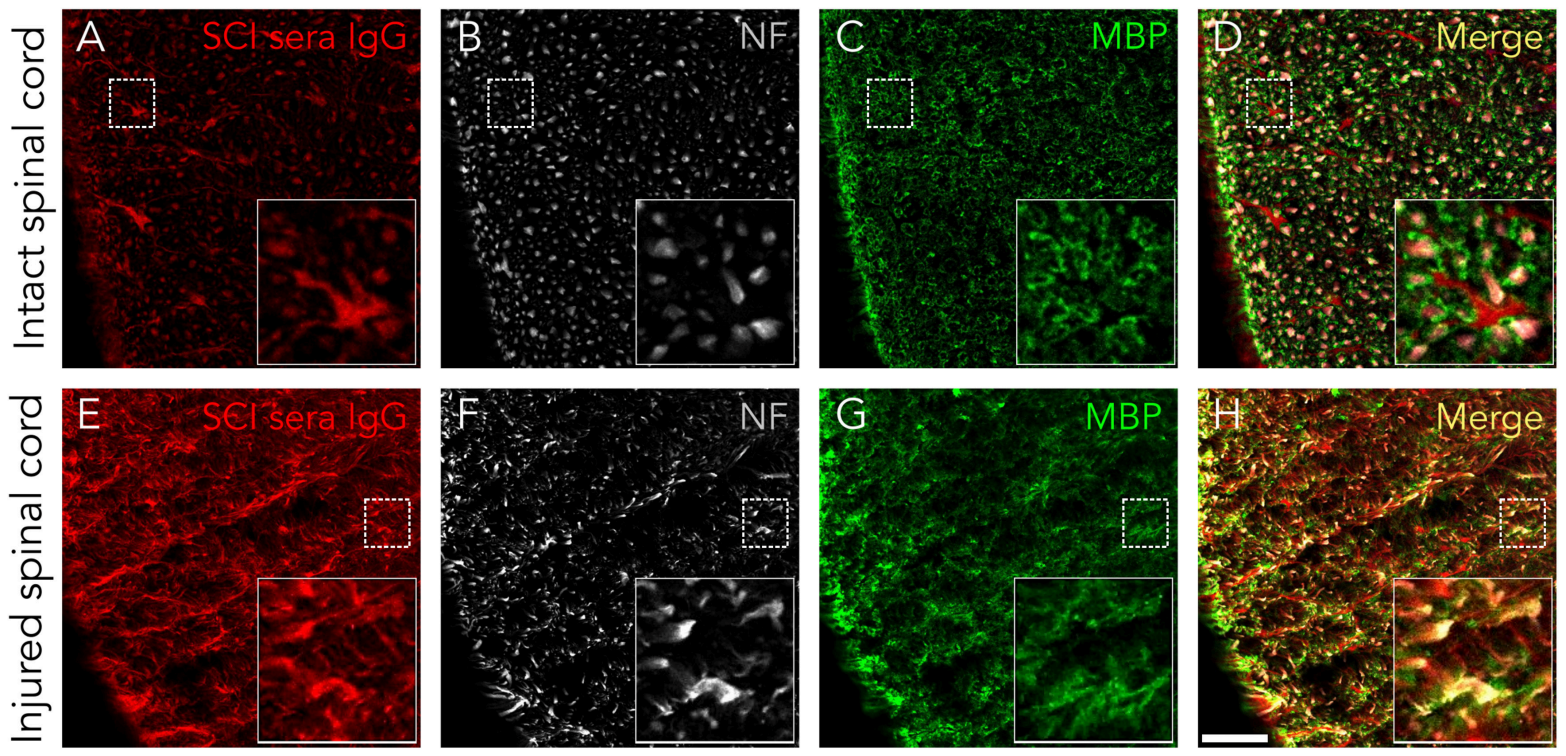
Serum IgGs react with antigens expressed on axons and disorganized myelin. IgGs from SCI individuals (in red) bind to normal appearing axons (NF-positive, in white) in control rat spinal cords **(A–D)** as well as to dystrophic axons in injured tissue **(E–H)**. However, IgGs do not seem to react with normally appearing myelin (as detected by being MBP-positive, in green), although they can be localized in some disorganized myelin structures in SCI tissue (yellow signal in **G, H**). All images are a single confocal plane. NF, neurofilament. MBP, myelin basic protein. Scale bar, 50 μm.

**Figure 3:**
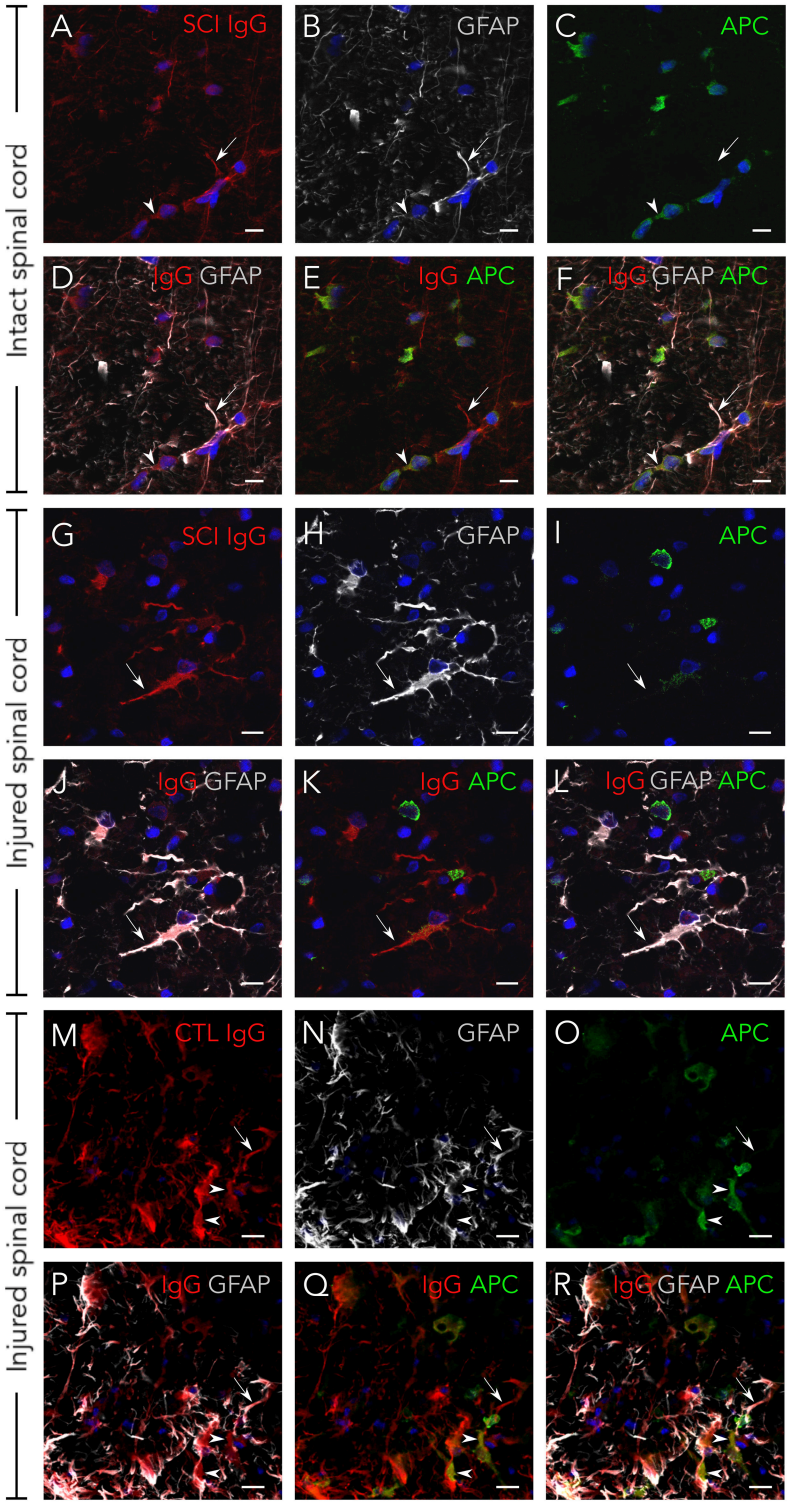
Serum IgGs react with antigens expressed on astrocytes and oligodendrocytes. IgG from SCI individuals (in red) bind to astrocytes (GFAP-positive, in white) and oligodendrocytes (APC-positive, in green) both on intact spinal cord tissue **(A–F)** and on injured tissue **(G–L)**. The same immunostaining pattern is observed with IgGs from healthy individuals on injured tissue **(M–R)**. As shown in the micrographs, not all astrocytes or oligodendrocytes in the rat spinal cord are targeted by human IgGs. All images are maximal projections obtained from a z-stack formed by five confocal planes. GFAP, glial fibrillar acidic protein. APC, adenomatous polyposis coli. Arrows point to IgGs reacting on astrocytes and arrowheads to IgGs reacting on oligodendrocytes. Scale bar, 10 μm for all images.

We minimized the number of rats subjected to SCI. In detail, three rats were lesioned to detect endogenous IgGs in the spinal cord at 1 day after injury by immunohistochemistry (results shown in Figure **5**); two to be employed in immunohistochemistry with human sera at 14 days after injury (results shown in Figures 1–**3**); two to isolate spinal cord proteins at 1 day after injury to be employed in Western blot experiments with human sera (results shown in Figure 1); and two to isolate spinal cord proteins at 28 days after injury to be used in Western blot experiments with human sera (results shown in Figure 1). In addition, two intact rats were sacrificed and perfused for immunohistochemistry experiments (results shown in Figures 1–**3**, **5**) and two other intact rats were sacrificed to isolate spinal cord proteins for Western blot experiments (Figure 1).

### Processing of rat spinal cord for immunohistochemistry

At 1 or 14 days after injury, rats were anesthetized with an intraperitoneal injection of sodium pentobarbital and transcardially perfused with 4% paraformaldehyde in 0.1 M phosphate buffer. The spinal cord was dissected out and post-fixed for 4 h in the same solution at 4°C. Tissue blocks of 1 cm were embedded in low-melting agarose and cut into 40 μm thick coronal sections with a Leica VT1000S vibratome (Madrid, Spain). Sections were stored in Olmos solution until they were used for free-floating immunohistochemistry.

### Inmunohistochemistry

Rat spinal cord was processed as previously described (31) using as primary antibodies the IgGs contained in sera samples or commercial antibodies against MBP (1:1,000, Covance, Princeton, NJ, USA), NFM (1:500, Aves Lab, Tigard, Oregon, USA), GFAP (1:2,000, Dako, Glostrup, Denmark) and APC (1:300, Calbiochem, Darmstedt, Germany). To detect these antibodies we used Cy3 conjugated anti-human IgG (1:1,000, Jackson Immunoresearch, Ely, UK), Cy5 anti-chicken IgY, Alexa Fluor-594 anti-rabbit IgG (1:1,000, Invitrogen, Barcelona, Spain) and Alexa Fluor-488 anti-mouse IgG (1:1,000, Invitrogen). To detect endogenous IgG in rat spinal cord, HRP-conjugated anti-rat IgG (1:1,000; Jackson Immunoresearch) was used and their localization was detected by DAB-peroxidase reaction. Also, HRP-conjugated anti-rabbit IgG (1:1,000; Jackson Immunoresearch) was employed as a control of specificity. When performed, nuclei were counterstained with Hoescht 33,258 (1:5,000; Invitrogen).

Samples were analyzed with a LEICA SP5 confocal microscope. All post-capture image modifications were identically performed between groups, including cropping, noise reduction and minor adjustments to optimize contrast and brightness.

### IgG and IgM enzyme-linked immunosorbent assays (ELISA)

Total levels of serum IgG and IgM were determined with Human IgG or IgM Platinum ELISA kits (eBioscience-Thermo Fisher Scientific, Madrid, Spain) following manufacturer's instructions. All samples were measured by duplicate and those with a coefficient of variation >10% were repeated. Dilution curves of standard IgG or IgM were performed for each 96-well plate. Consistency of results among different plates was confirmed by introducing the same sample from a healthy control subject in all plates and observing reproducible values. When the values of this sample were not reproducible, the whole plate was discarded and repeated.

### Protein isolation from human and rat spinal cord

Protein isolation was performed with ReadyPrep™ protein extraction kit for soluble and insoluble proteins (Bio-Rad, Madrid, Spain). Briefly, tissue was mechanically homogenized and sonicated in chilled 40 mM Tris base buffer supplemented with protease inhibitors (Complete Mini, Roche Diagnostics, Mannheim, Germany). The volume of buffer employed for every tissue sample was escalated to be in the range of 200 mg of tissue per 1 ml of buffer. Samples were centrifuged at 15.000 × g for 30 min at 4°C and supernatants –fraction containing hydrophilic proteins– were collected. Pellets were resuspended and sonicated in the same volume of 2-D rehydration/sample buffer, containing 7 M urea, 2 M thiourea, 1% ASB-14 detergent, 40 mMTris base and 0.001% bromophenol blue supplemented with 0.2% tributylphosphine (TBP) reducing agent. Samples were centrifuged at 15,000 g for 20 min at RT and supernatants –fraction containing hydrophobic proteins– were collected. Protein concentration of both insoluble and soluble fractions was determined by RC DC protein assay (Bio-Rad). Samples were aliquoted and stored at −80°C until further use.

In the case of rat spinal cord, intact and injured rats (at 1 and 28 days after lesion) were anesthetized with an intraperitoneal injection of sodium pentobarbital. The spinal cords were dissected out at 4°C and tissue blocks of 5 mm comprising the rostral part of the lesion epicenter were processed for protein isolation. Protein isolation and determination of their concentration was performed as described above, and samples were aliquoted and stored at −80°C until further use.

### Western blot with rat spinal cord tissue

Protein lysates were mixed with 5× Laemmli sample buffer and heated at 60°C for 5 min. Equal amounts of protein were resolved in 4–15% gradient Mini-PROTEAN® TGX™ precast protein gels (Bio-Rad) and electroblotted onto nitrocellulose membranes. Membranes were blocked for 30 min at room temperature with synthetic blocking solution (Invitrogen, Madrid, Spain) and incubated overnight at 4°C with a pool of human sera (1:500 dilution) from control or SCI individuals. Pools were constructed by mixing equal volumes of sera from four different subjects. Three different serum pools of intact individuals and three different pools of SCI patients were used. Membranes were then incubated at room temperature for 90 min with Alexa Fluor 790-conjugated anti-human IgG antibodies (Fc_γ_ fragment specific, 1:1,000; Jackson Immunoresearch). Protein loading was evaluated by incubating the membranes overnight at 4°C with anti-GAPDH antibody (1:500,000; Abcam, Cambridge, UK) followed by incubation with secondary Alexa-680 anti-mouse IgG (1:5,000; LI-COR Biosciences) for 90 min at room temperature. Blots were visualized and acquired using Odyssey® CLx equipment (LI-COR Biosciences, Lincoln, NE, USA).

Immunoreactivity of human serum IgGs against spinal cord proteins from intact or injured rats was quantified by determining the integrated intensity of the protein bands observed in each western blot (WB) lane using the FIJI distribution of ImageJ software (32). The sum of integrated intensities for a given lane was normalized to the protein loading in that lane estimated by measuring GAPDH integrated intensity.

### Detection of human spinal cord targets of autoantibodies by two dimensional-western blot

Due to the lack of non-fixed spinal cord samples from SCI patients and considering that autoantibodies could be directed against proteins newly expressed or modified by lesion, we searched for human spinal cord samples where inflammation- or cell death-induced modifications could be present. Therefore, we pooled proteins extracted from pathological spinal cord tissue obtained from Balo concentric sclerosis lesions, multiple sclerosis demyelinated areas and amyotrophic lateral sclerosis. In addition, since it could not be discarded that some autoantibodies might be directed against proteins more abundantly or exclusively present in control subjects, we also extracted proteins from non-pathological human spinal cord samples and mixed them in a final pool with pathological spinal cord tissue samples (Supplementary Table 1). Frozen non-fixed human spinal cord tissue blocks were kindly supplied by the Neurological Tissue Biobank of Hospital Clinic-IDIBAPS (Barcelona, Spain) and CIEN Foundation Tissue Bank (BT-CIEN; Madrid, Spain). Protein isolation was performed as described above. Proteins were precipitated using the 2D Clean Up Kit (Sigma, Madrid, Spain) following manufacturer's instructions and resuspended in labeling buffer containing 7 M urea, 2 M thiourea, 4% CHAPS and 30 mM Tris. After checking samples to be between pH 8.0–9.0, soluble and insoluble fractions were mixed in a 1:1 ratio. Proteins were then labeled with Cyanine 2 NHS ester minimal dye (Lumiprobe, Hannover, Germany) following manufacturer's instructions, for 30 min in dark conditions.

Non-lineal 11 cm strips, pH 3–10 (GE Healthcare, Little Chalfont, UK), were placed in a re-swelling tray (GE Healthcare) to actively rehydrate overnight with 150 μg Cy2-labeled proteins. Isoelectric focusing was then performed in a Protean IEF cell unit (Bio-Rad), at 20°C, according to the following program: 30 min at 500 V, 1 h at 1,000 V (ramping), 3 h at 5,000 V (ramping) up to a total of 35,000 V/h. After isoelectrofocusing, strips were equilibrated in 1.5 M Tris, ph 8.8 buffer containing 6 M urea, 30% glycerol, 2% SDS and bromophenol blue, with the addition of 1% DTT for 20 min. Then, strips were incubated for 20 more min in the same buffer in the presence of 2.5% iodoacetamide. Proteins were then separated accordingly to their molecular weight in Criterion™ TGX™ AnykD™ midi precast gels (Bio-Rad).

After 2D-electrophoresis, proteins were transferred to low fluorescence PVDF membranes with a semi-dry protocol using Trans-Blot® Turbo™ transfer system (Bio-Rad). Membranes were blocked with BlockAid solution (Thermo Fisher Scientific, Madrid, Spain) for 30 min at RT and incubated overnight at 4°C with sera diluted 1:500 in PBS, 0.1% Triton X-100 and 10% BlockAid Solution. After several washing steps, membranes were incubated for 2 h at RT with secondary Cy3-conjugated goat anti-human IgG (Fc_γ_ fragment specific; 1:1,000; Jackson Immunoresearch) and Alexa-647 goat anti-human IgM (Fc_5μ_ fragment specific; 1:1,000; Jackson Immunoresearch).

We performed the complete procedure described above for every serum sample analyzed (no stripping and reblotting of membranes was performed). Reproducibility of the procedure was tested by randomly repeating five serum samples and confirming in a selected subset of protein spots that bound AAb values were similar between duplicates.

### 2D-WB images acquisition

2D-WB were visualized with the laser confocal scanner Typhoon™ Trio (GE Healthcare) and images were acquired at 50 μm/pixel with a 16-bit depth. Voltage of photomultipliers was maintained throughout all experiments. To determine voltage values for Cy2 channel –the whole spinal cord proteome– we confirmed that no signal was observed in a membrane without Cy2-labeled protein and we fixed a value that did not result in a saturated signal. For Cy3 and Alexa-647 channels we confirmed that no signal was detected when omitting the secondary antibodies and we fixed values that did not result in saturation.

### 2D-WB analysis

Protein spots resulting from 2D-WB were segmented and matched among membranes with DeCyder 7.0 software (GE Healthcare). A careful visual inspection of segmentation and matching was performed and errors were manually corrected. Also, spots containing technical artifacts, like air bubbles or fluorochrome precipitates, were eliminated. After background correction, the volumes (integrated densities) of every spot on Cy2, Cy3 and Alexa-647 channels were exported and processed with R programming language (33). Minimal labeling with Cy2 dye ensures labeling of a single lysine per protein molecule, so Cy2 integrated density values are proportional to protein loading. Cy3 integrated density –bound IgG AAb– and Alexa-647 integrated density –bound IgM AAb– are a function of their abundance and of that of their antigenic target. Therefore, every Cy3 and Alexa-647 values were corrected by the Cy2 value of their respective target. All values were transformed to log_2_ scale, so all IgG and IgM level values are the log ratio of their abundance relative to their antigenic target abundance:

$$\begin{array}{l} IgG\;AAb=\log_{\begin{array}{c} 2 \end{array}}\left(\frac{Cy3\;integrated\;density}{Cy2\;integrated\;density}\right)\\ IgM\;AAb=\log_{\begin{array}{c} 2 \end{array}}\left(\frac{Alexa\text{\_}647\;integrated\;density}{Cy2\;integrated\;density}\right) \end{array}$$

### Comparison of AAb binding to their targets between SCI and control individuals

In every 2D-WB performed, any spinal cord protein recognized by IgG and/or IgM was annotated. This procedure was performed with all control and SCI individuals, generating a list of all antigenic targets visually detectable by AAbs in our population. Binding of AAb to their targets were compared between SCI and control subjects by two-tailed *t*-test. Multiple comparison correction was performed by Benjamini-Yekutieli method (34). All statistical analysis were performed in RStudio (35).

### Identification of antigenic targets by MALDI/MS-MS

Targets of AAbs were in-gel digested using the Ettan Digester (GE Healthcare). The digestion protocol used was that of Shevchenko et al. (36) with minor variations: gel plugs were submitted to reduction with 10 mM dithiothreitol (Sigma Aldrich) in 50 mM ammonium bicarbonate (99% purity; Scharlau) and alkylation with 55 mM iodoacetamide (Sigma Aldrich) in 50 mM ammonium bicarbonate. The gel pieces were then rinsed with 50 mM ammonium bicarbonate in 50% Methanol (gradient, HPLC grade, Scharlau) and acetonitrile (gradient, HPLC grade, Scharlau) and dried in a Speedvac. Modified porcine trypsin (sequencing grade; Promega, Madison, WI, USA) at a final concentration of 20 ng/μl in 20 mM ammonium bicarbonate was added to the dry gel pieces and the digestion proceeded at 37°C overnight. Finally, 60% aqueous acetonitrile and 0.1% trifluoroacetic acid (99.5% purity; Sigma Aldrich) were added for peptide extraction. To confirm protein identity, digestion of every spot was repeated at least from two different gels. Then, proteins were identified by MALDI/MS-MS: 0.5 μl of each digestion solution were deposited using the thin layer method, onto a 384 Opti-TOF 123 × 81 mm MALDI plate (Applied Biosystems, Madrid, Spain) and allowed to dry at room temperature. The same volume of matrix (3 mg/mL α-cyano-4-hydroxycinnamic acid –Sigma Aldrich– in 60% acetonitrile, 0.1% trifluoroacetic acid) was applied on every sample in the MALDI plate. Samples were deposited by duplicate in every MALDI plate to avoid cross-contamination. MALDI-MS/MS data were obtained in an automated analysis loop using a 4800 Plus MALDI TOF/TOF Analyzer (Applied Biosystems). Spectra were acquired in the reflector positive-ion mode with a Nd:YAG, 355 nm wavelength laser, at 200 Hz laser frequency, and 1,000–2,000 individual spectra were averaged. The experiments were acquired uniform with fixed laser intensity. For MS/MS 1 kV analysis mode, precursors were accelerated to 8 kV in source 1, selected with a relative resolution of 200 (FWHM) and metastable suppression. Fragment ions generated by collision with air in a CID chamber were further accelerated by 15 kV in source 2. Automated analysis of mass data was performed using the 4000 Series Explorer Software version 3.7.0 (Applied Biosystems). Internal calibration of MALDI-TOF mass spectra was performed using two trypsin autolysis ions with m/z = 842.510 and m/z = 2,211.105. For MALDI-MS/MS, calibrations were performed with fragment ion spectra obtained for Glub-fibrinopeptide (4700 Cal Mix, Applied Biosystems). MALDI-MS and MS/MS data were combined through the ProteinPilot Version 5.0.1 to search a nonredundant protein database (Swissprot 2017_02) using the Mascot software version 2.5 (Matrix Science) with 50 ppm precursor tolerance, 0.6 Da MS/MS fragment tolerance, CAM (carbamidomethylcystein) as fixed modification, oxidized methionine as variable modification and allowing 1 missed cleavage. MALDI-MS/MS spectra and database search results were manually inspected in detail using the aforementioned software.

### Maps of protein isoforms targeted by AAbs

The observed molecular weight (MW) and isoelectric point (pI) of the proteins targeted by AAbs were calculated with the Biological Variation Analysis module implemented in DeCyder 7.0 software (GE Healthcare). The predicted MW of the different alternative splicing isoforms of each protein were obtained from UniProtKB database (37) and their pIs were calculated with ExPASy's compute pI/MW tool based on their aminoacidic sequence (38, 39). Observed protein targets of AAbs are represented by circles and the predicted basal isoforms by crosses in order to allow a rapid visual estimation of how much AAb-targeted isoforms differ from their basal (non-modified) proteins. All maps were represented in RStudio.

### Functional enrichment analysis

Cytoscape (40) app ClueGO (41) was used to perform functional enrichment analysis of AAb targets. Human Gene Ontology Biological Function database from 18.05.2017 was used. Two-tailed hypergeometric test followed by Benjamini-Hochberg multiple comparison correction was employed. Only terms significantly enriched after multiple comparison correction (*p* < 0.05 and false discovery rate (FDR) < 0.05) are shown in the results section.

### Other statistical analysis

Comparison of IgG binding from healthy and SCI individuals to proteins isolated from intact and injured spinal cord (shown in Figure 1N) was performed by two-way ANOVA in R Studio, using stat package (33).

## Results

### IgGs against proteins found in damaged spinal cord are present in the sera from healthy individuals and their levels increase in SCI patients

Serum IgG levels in SCI patients are significantly higher at 1 month after injury compared to healthy subjects, while IgM levels fail to reach statistical significance (Figures 1A,B). In experimental models, at the acute phase, total IgG and IgM serum levels increase after low thoracic lesions (3) and decrease after cervical lesions (16). Contrasting with these observations, in our study neither neurological level of injury nor severity affects total IgG or IgM serum levels (Figures 1C–F). To explore whether the increased serum IgG after SCI could be related to higher levels of autoantibodies (AAbs), we tested the immunoreactivity of sera samples IgG against rat spinal cord tissue. Confocal microscope settings were tuned to assure that any signal detected was specific, not attributable to background or unspecific staining from secondary antibodies. For this purpose, sections from rat spinal cord at 14 days after lesion were incubated only with the secondary anti-human IgG Ab used to detect IgG in human sera. This control immunostaining does not display any signal neither around the lesion epicenter when photomultiplier gain is set up at 750 mV, nor in the lesion epicenter (where autofluorescence is higher) with even less restrictive conditions (gain at 850 mV; Figures 1G–H). On the contrary, at the lower photomultiplier sensitivity setting −750 mV– specific signal is observed whenever human serum is tested (Figures 1I–L). Indeed, a weak although specific IgG signal is detected with sera from healthy control subjects on intact rat spinal cord, depicting mainly motor neurons in the ventral horn (Figure 1I). However, when sera from control subjects are incubated with injured spinal cord tissue sections instead of intact tissue, an unexpected stronger IgG signal against spinal cord antigens is observed (Figure 1K). In this case, cellular structures are observed consistent with neuronal bodies in the gray matter and axons and glia in the white matter. In comparison, with the same confocal settings, sera from SCI patients show a robust IgG signal against intact rat spinal cord, depicting motor neurons in the ventral horn as well as axons and glial profiles in the white matter (Figure 1J), which among other mechanisms could be related to the higher IgG levels in SCI sera (Figure 1A). Also, IgG from SCI patients strongly bind to injured spinal cord tissue (Figure 1L). Altogether, our results suggest that both healthy and SCI individuals carry IgG AAbs against spinal cord antigens, although AAb from healthy individuals are clearly detectable only when their sera are tested against injured spinal cord. To confirm this, we analyzed by western blot the binding of serum IgGs to proteins isolated from spinal cords of intact and injured rats at 1 and 28 days after lesion (Figure 1M). Our results show that IgGs from healthy control subjects display the same pattern of bands against protein extracts from both intact and injured rat spinal cords, although immunoreactivity is stronger against proteins isolated from injured spinal cord (Figure 1M, left panel). Similarly, IgGs from SCI patient's sera recognize the same pattern of bands in protein extracts from both intact and injured spinal cord (Figure 1M, right panel). Two-way ANOVA of Western blots quantification confirms that IgG immunoreactivity from SCI patients is significantly higher than that of IgGs from control individuals (*p* = 0.006; Figure 1N). Also, Student's *t*-tests confirms that IgG immunoreactivity from control subjects is significantly higher toward proteins isolated from injured spinal cords at 1 or 28 days after injury than toward proteins isolated from intact spinal cords (Figure 1N).

### Serum IgGs target antigens expressed on neurons, astrocytes, and oligodendrocytes

Although serum IgGs clearly depict ventral horn motoneurons and white matter axons (Figure 1), we confirmed this by immunohistochemistry: Figure 2 shows that IgGs from SCI patients bind to cellular structures positive for Neurofilament (NF) both in intact and injured spinal cord white matter. Interestingly, while IgGs from SCI patients do not bind to intact myelin surrounding white matter axons [determined as structures positive for Myelin Basic Protein (MBP)], they react with disorganized myelin adjacent to dystrophic axons on injured spinal cords (Figures 2C,D,G,H). Also, IgGs bind to cellular profiles negative for NF and MBP: as shown in Figure 3, patient's IgGs bind to cells expressing Glial Fibrillar Acidic Protein (GFAP, a marker of astrocytes), and Adenomatous Polyposis Coli (APC, a marker of oligodendrocytes) both in tissue sections of intact (Figures 3A–F) and injured spinal cord (Figures 3G–L). IgGs from healthy subjects also bind to neurons (see ventral horn motoneurons depicted in Figure 1K), astrocytes and oligodendrocytes in the injured spinal cord (Figures 3M–R).

Altogether, our results show that increased AAbs after SCI target neurons, astrocytes and oligodendrocytes in the spinal cord.

### Autoantibodies increased at the acute phase are naturally occurring antibodies preferentially directed against non-native isoforms of 16 different proteins

**Figure 4:**
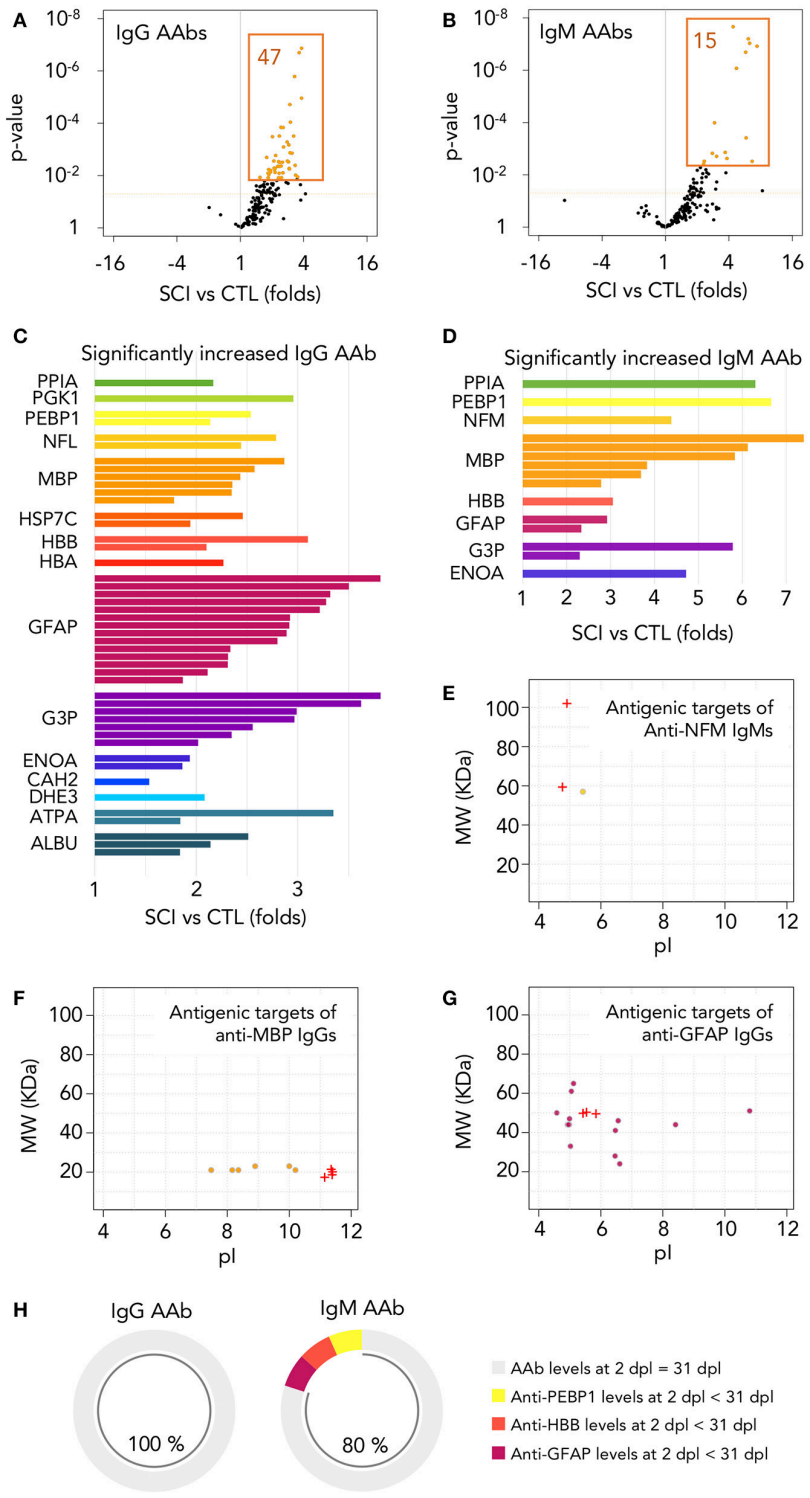
Autoantibodies against modified central nervous system and peripheral proteins are rapidly increased after injury. **(A,B)** Among the 173 different IgG and IgM autoantibodies detected, 47 IgGs and 15 IgMs are significantly increased at 1 month after injury (*t*-test *p*-value < 0.05 and FDR < 0.05 after multiple comparison correction by Benjamini-Yekutieli method). Dotted horizontal orange line represents *t*-test *p*-value = 0.05. Orange-filled spots represent FDR < 0.05 after multiple comparison correction by Benjamini-Yekutieli method **(C,D)** Relative abundance and identity of the 47 IgGs and 15 IgMs increased in SCI patients. PPIA, peptidylprolyl isomerase 1; PGK1, phosphoglycerate kinase 1; PEBP1, phosphatidylethanolamine binding protein 1; NFL, neurofilament light; NFM, neurofilament intermediate; MBP, myelin basic protein; HSP7C, heat shock cognate 71 KDa protein; HBB, hemoglobin subunit beta; HBA, hemoglobin subunit alpha; GFAP, glial fibrillar acidic protein; G3P, glyceraldehyde-3-phosphate dehydrogenase; ENOA, alpha-enolase; CAH2, carbonic anhydrase 2; DHE3, glutamate dehydrogenase 1, mitochondrial; ATPA, ATP synthase subunit alpha, mitochondrial; ALBU, albumin. **(E–G)** Most of the autoantibodies increased after SCI are directed against proteins with modifications affecting their isoelectric point (pI) and/or their molecular weight (MW). Crosses represent the pI and MW of the basal isoforms produced by alternative splicing and circles represent the isoforms targeted by autoantibodies increased after injury. **(H)** Among the 47 IgGs increased at 1 month after injury, all of them reached similar levels at 2 days after injury (no statistically significant differences were found after paired *t*-test between AAb binding at both times), while the same is observed for 12 out of the 15 IgMs.

We detected and quantify the binding of serum AAbs to their targets by Two Dimensional-Western blot (2D-WB) of sera against a pool of proteins extracted from pathological and control human spinal cords. Afterwards, we identified AAb targets by mass spectrometry (workflow schematized in Supplementary Figure 1). Thus, by 2D-WB we observed, again, that AAbs are present in the sera of both control and SCI subjects (Supplementary Figure 2) and bind to 173 different targets that correspond to isoforms/post-translational modifications of 37 different proteins (Supplementary Table 2). An overall increase in both IgG and IgM AAb levels after SCI compared with age- and gender-matched control individuals is observed (volcano plot representations of SCI/control ratios are heavily skewed toward the right; Figures 4A,B), although only 47 IgG and 15 IgM AAbs reached statistical significance (*t*-test followed by multiple comparison correction by Benjamini-Yekutieli method under a false discovery rate (FDR) minor than 0.05; orange spots in Figures 4A,B). The increased IgGs and IgMs react against several isoforms/post-translational modifications of a limited number of proteins. Specifically, 15 different proteins are targets of IgG (Figure 4C) while 8 are targets of IgMs (Figure 4D). Seven out of eight targets of IgMs are also targeted by IgGs, so a total of 16 different IgG and IgM AAbs specificities are detected. Among these, anti-GFAP, -MBP and -G3P AAbs have been previously described to increase after SCI, but the remaining AAbs are, to our knowledge, for the first time described to augment in this study. Identification of AAbs against GFAP, MBP, neurofilament light (NFL) and neurofilament intermediate (NFM) confirms our immunohistochemical observations; i.e., astrocytes, oligodendrocytes and neurons are targets of AAbs after SCI. On the other hand, we have also identified targets whose expression is not restricted to the central nervous system [for example albumin (ALBU), hemoglobin subunit alpha (HBA) or hemoglobin subunit beta (HBB)]. The antigenic targets recognized by AAbs for which there are not significant differences between healthy and SCI individuals are shown in Supplementary Table 2 and include actin cytoplasmic 1 (ACTB), fructose-biphosphate aldolase C (ALDOC), annexin A2 (ANXA2), annexin A5 (ANXA5), carbonyl reductase 1 (CBR1), 60 kDa heat shock protein (CH60), dihydropyrimidinase-related protein 2 (DPYL2), stress-70 protein (GRP75), heat shock-related 70 kDa protein 2 (HSP72), malate dehydrogenase (MDHC), moesin (MOES), nicotinamide riboside kinase-2 (NRK2), peripherin (PERI), peroxiredoxin-1 (PRDX1), peroxiredoxin-6 (PRDX6), phosphoglycerate mutase 1 (PGAM1), pyruvate kinase (KPYM), tubulin alpha-1C chain (TBA1C), tubulin beta-4A chain (TBB4A), tubulin beta chain (TBB5), and vimentin (VIME).

Consistent with the description of natural autoantibodies as directed against modified proteins, most of the 47 IgGs and 15 IgMs increased after SCI bind to isoforms that differ in their isoelectric point (pI) and/or molecular weight (MW) from the basal predicted values (Figures 4E–G; Supplementary Figure 3), suggesting that most of the AAbs that we have detected in control and SCI subjects are directed against proteins with alterations or post-translational modifications. In this regard, some AAbs elevated after SCI recognize proteins with a lower MW than predicted, as occurs with the increased anti-NFM IgM AAb, that recognize an isoform around 60 kDa whose peptidic sequence confirms that is a degraded fragment of the canonical 102 kDa NFM (Figure 4E). Other AAbs recognize modifications of targets that affect their pI, as anti-MBP IgGs, that are directed against more acidic forms than the native isoforms (Figure 4F), and other AAbs recognize isoforms with alterations affecting both their MW and pI, as occurs with the elevated anti-GFAP IgGs (Figure 4G; MW and pI of all other antigenic targets shown in Supplementary Figure 3). Overall, our results suggest that the AAbs increased after SCI are directed against degraded protein fragments and/or post-translational modifications of proteins.

If as our results suggest, AAbs increased after SCI are natural antibodies (AAb naturally present in healthy subjects), their augmentation should be the result of a secondary humoral response and, consequently, their increase should be detected very early after SCI. To test this, we had the opportunity of assessing sera at 2 ± 0.4 days after injury (mean and standard error) obtained from a subgroup of 11 patients among the 52 individuals studied at 1 month. We compared in these patients by paired *t*-test the AAb levels at 2 days with those at 1 month after injury. As shown in Figure 4H, all 47 IgG augmented at 1 month after injury are already increased at 2 days (no statistically significant differences are found between 1 month and 2 days), and the same is observed for 12 out of 15 IgMs (80%), supporting that most AAb increased at the acute phase of SCI are natural antibodies.

### Endogenous IgG AAbs against spinal cord antigens are also detected very early after experimental SCI in rats

**Figure 5:**
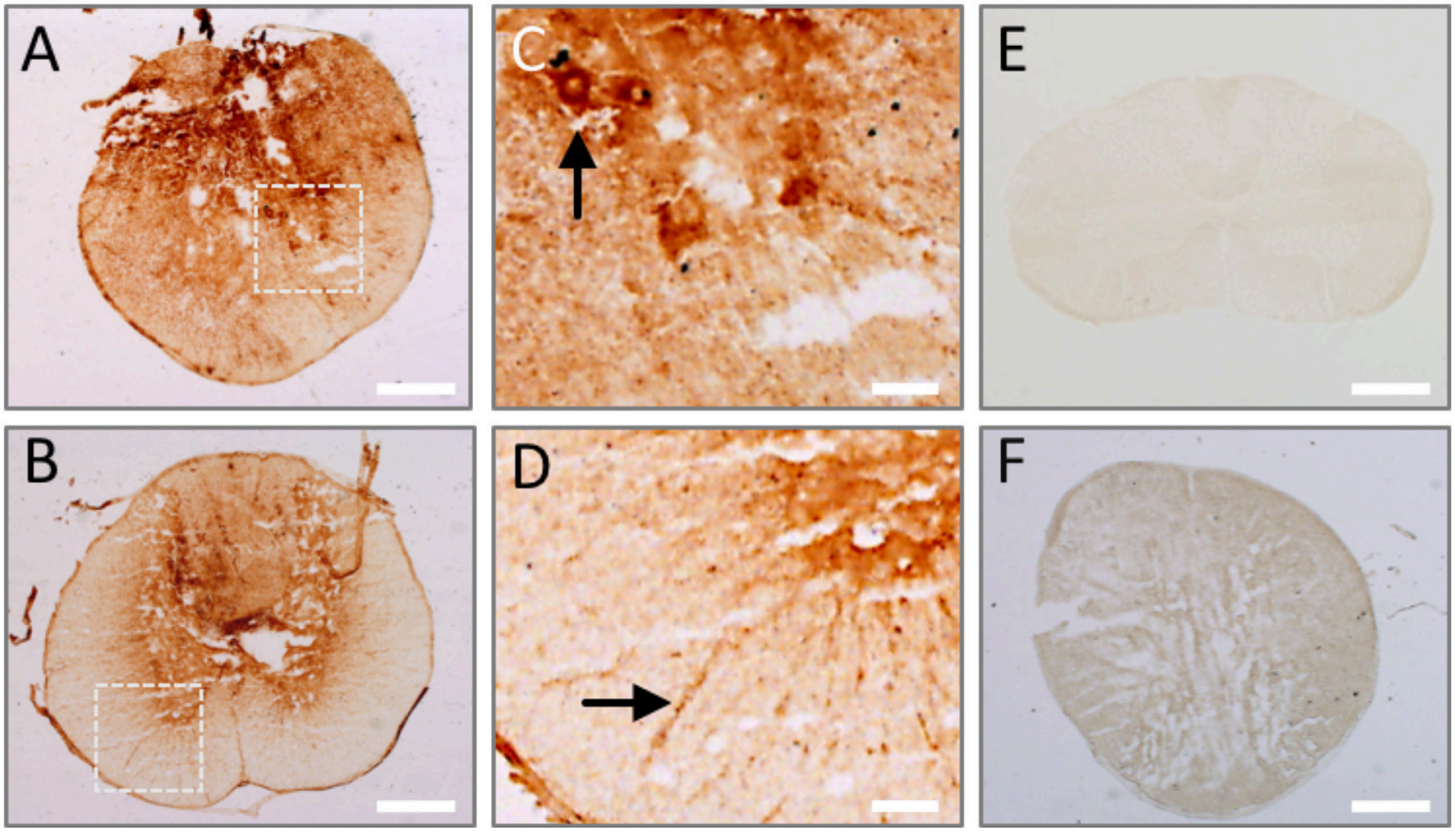
Endogenous IgGs are detected in the spinal cord at 1 day after injury. Tissue sections from rat spinal cord at 1 day after injury were incubated with anti-rat IgG antibody and its binding was revealed by DAB-peroxidase reaction. Endogenous rat IgG is detected and depicts cellular profiles both in the lesion epicenter **(A)** and periphery **(B)**. **(C,D)** Show magnified details of inserts in **(A,B)**, where cell profiles in the ventral horn (arrow in **C**) and white matter (arrow in **D**) are clearly marked by endogenous IgG. **(E)** No endogenous IgG is observed in intact spinal cord. **(F)** IgG signal is species-specific, as shown by the absence of immune detection when using a secondary anti-rabbit IgG on injured spinal cord. Scale bar, 500 μm in **(A,B,E,F)**; 100 μm in C and D.

Since our results show that AAbs against injured spinal cord are naturally present in the sera of healthy subjects, theoretically they should extravasate into the spinal cord once the blood-spinal cord barrier breaks after SCI and should be detectable at early stages, before new IgGs may be synthesized. We experimentally confirmed this in rats, observing endogenous IgGs in the injured spinal cord after 1 day, depicting cellular profiles at the lesion epicenter (Figure 5A) and on the most rostral spinal cord level affected by the lesion (Figure 5B). Target cells are located both in the gray matter (as the cell profile pointed out in the ventral horn in Figure 5C) and in the white matter (Figure 5D). However, as expected, endogenous IgGs cannot be detected in the spinal cord of intact rats (Figure 5E) and no signal is observed when tissue sections are incubated with an antibody against rabbit-IgG (signal is species-specific; Figure 5F). Therefore, in agreement with the results described above on AAbs after human SCI, naturally occurring IgG AAbs against injured spinal cord are also detectable in rodents.

### Binding of autoantibodies to spinal cord proteins increases after injury independently of lesion level and severity

**Figure 6:**
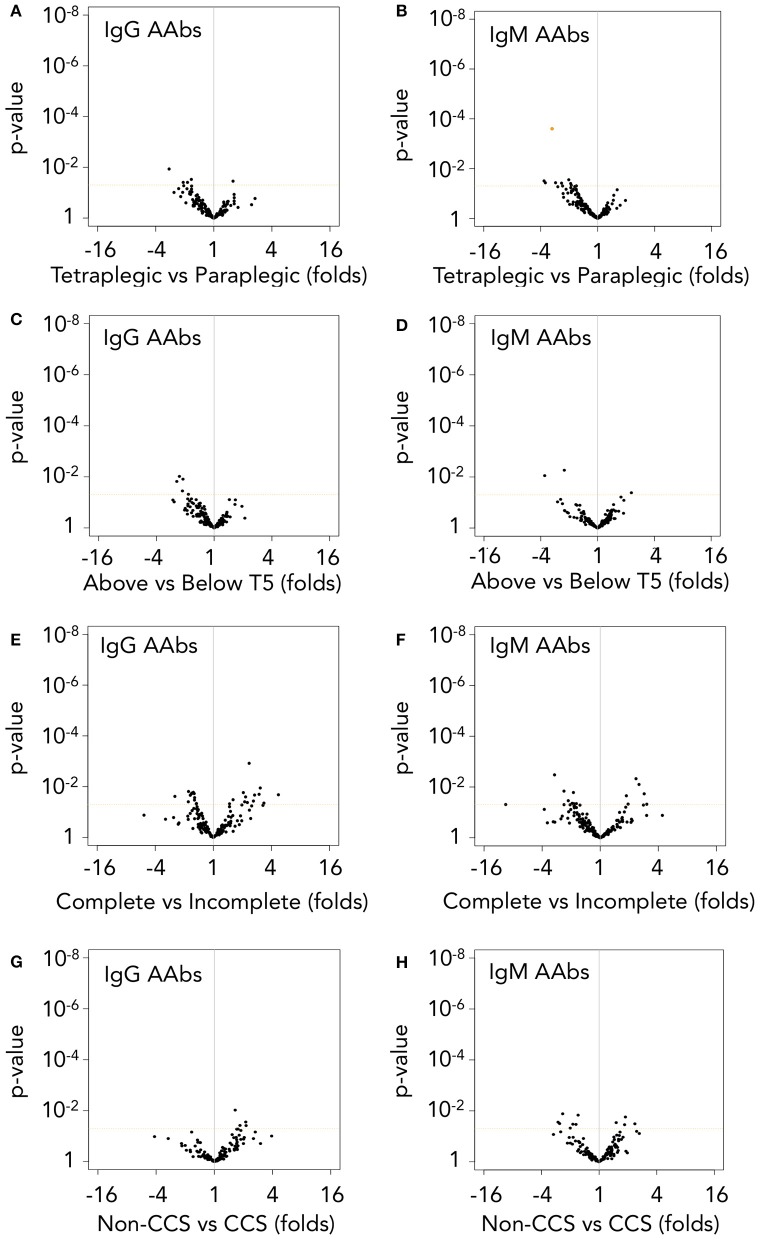
Autoantibody levels are higher in spinal cord injury patients independently of lesion level and severity. **(A,B)** IgG and IgM AAb binding are not significantly different after performing *t*-test (*p*-value in *y*-axis) followed by false discovery rate (FDR) correction between tetraplegic and paraplegic patients, but for a single IgM AAb against HBA (orange-filled spot, **B**). **(C,D)** However, when classifying patients into those with lesions above or below T5 spinal segment, not a single AAb present different levels between the two groups. **(E,F)** Complete patients (AIS **A**) and incomplete patients (AIS **B–D**) do not show significant differences in IgG nor IgM AAb levels. **(G,H)** Comparison of cervical AIS D central cord syndrome patients (CCS, a less severe traumatic spinal cord injury) with their counterparts cervical AIS A patients (Non-CCS) also fails to found any statistically significant difference in AAb levels due to lesion severity. Dotted horizontal orange line represents *t*-test *p*-value = 0.05. Orange-filled spot represents FDR < 0.05 after multiple comparison correction by Benjamini-Yekutieli method.

Cervical or high thoracic lesions impairs primary humoral responses to a greater extent than low thoracic or lumbar lesions (8, 15). However, secondary immune responses are not affected by injury level (17). Our results show that binding of IgG and IgM AAb to their targets is not affected by lesion level: only 1 IgM AAb against hemoglobin subunit alpha (HBA) is increased in paraplegics compared with tetraplegic patients (*t*-test *p*-value < 0.5 and FDR < 0.05, Figures 6A,B) while not a single AAb is significantly affected by lesion level when classifying patients based on whether lesion is above or below the 5th thoracic segment (Figures 6C,D).

Also, in contrast with previous reports, the severity of lesion does not have an overall effect on IgG or IgM AAb binding to their targets (Figures 6E,F). To further assess the effect of severity on AAbs after SCI, we analyzed whether AAb levels were different in a less severe SCI group, constituted by ten cervical AIS D central cord syndrome patients (CCS) (42). In fact, with the exception of slight anemia and thrombocythemia, the hematological values of CCS patients are like those of control subjects, while their counterparts cervical AIS A non-CCS SCI patients present more severe alterations that also affect to leukocyte populations (Supplementary Table 3). However, the levels of AAbs of CCS patients are like those of cervical AIS A non-CCS SCI patients (Figures 6G,H), reinforcing the observation that AAb levels are not affected by lesion severity.

### Functional enrichment analysis of autoantibody targets

**Figure 7:**
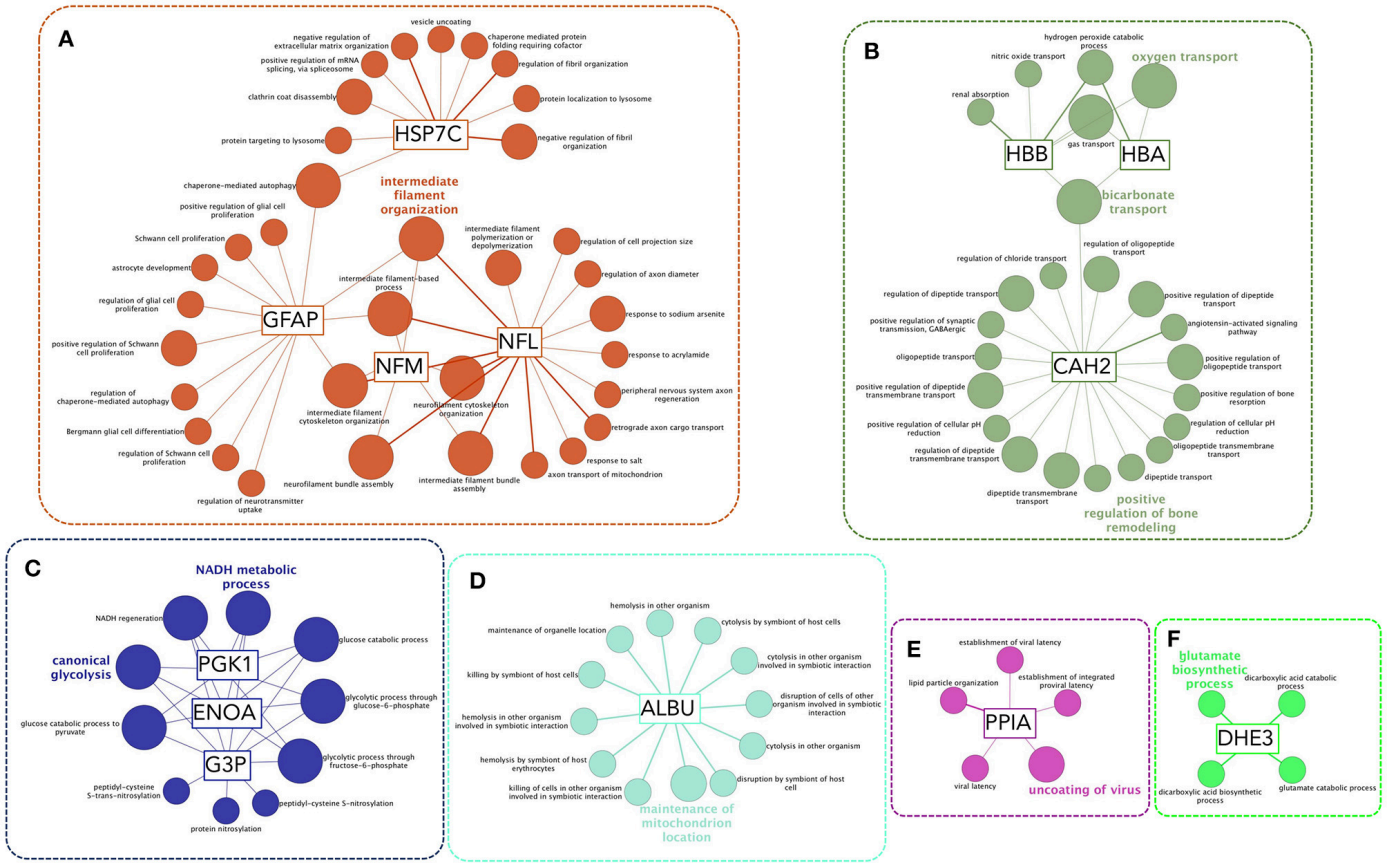
Functional enrichment analysis of autoantibody targets reveals functions that could be affected by AAbs or that are affected by other injury-related processes and are being sensed by AAbs. Biological Function Gene Ontology terms associated to AAb targets are functionally organized into a network structure. Only significant terms after hypergeometric test followed by Benjamini-Hochberg multiple comparison correction are shown. Most significant terms of each functional group are highlighted. Six functional clusters may be established **(A–E)**: **(A)** a cluster formed by terms coupled to intermediate filaments-related functions, including proliferation and autophagy; **(B)** a cluster related to the control of oxygen radicals and oxygen transport, that is functionally related to a subcluster comprising CAH2 (carbonic anhydrase 2) functions, including bone remodeling; **(C)** a cluster including terms related to energetic metabolism, comprising glycolysis and NAD and ATP biosynthesis; **(D)** functions related to albumin; **(E)** functions related to PPIA (also known as cyclophilin A) that are involved in control of viral latency; **(F)** glutamate biosynthesis.

AAbs increased after SCI might be involved in pathophysiology (5) and/or might serve as biomarkers of underlying pathophysiological alterations (43, 44). To gain insight into the biological role of the increased AAbs, we analyzed the gene ontology biological function terms associated to their targets. This results in the statistically significant over-representation of functions that can be clustered in six major groups: (1) functions related with cytoskeleton organization (intermediate filaments, Figure 7A); (2) oxygen transport and removal of oxygen reactive species (Figure 7B); (3) energetic metabolism (Figure 7C); (4) albumin-related functions (Figure 7D); (5) response to virus (Figure 7E) and glutamate biosynthesis (Figure 7F).

## Discussion

Our results show that SCI induces a rapid increase in the levels of pre-existing natural IgG and IgM AAbs against modified isoforms from 16 different proteins independently of lesion level and severity. Among the antigenic targets, 13 are described for the first time and could become new therapeutic targets to test in future studies. Altogether, our results suggest that the origin of antibody-mediated autoimmune responses after SCI is the expansion of previously formed natural AAbs.

### Autoantibodies that increase after SCI exist before lesion

Natural AAbs are usually overlooked in healthy population mainly due to technical reasons (45). In this regard, several methodological details may explain why we observe a specific signal from AAbs in healthy individuals. For instance, our use of immunofluorescence and high sensitivity scanning to detect AAbs may uncover signals that remain below the threshold of detection by commonly techniques like chemoluminiscence or peroxidase reactions. But a major difference between our study and others may be the antigen collection employed to test for AAbs levels and specificity on 2D-WB experiments. Since we observed that sera from healthy individuals contain AAbs that are preferentially directed against antigens present in injured tissue (Figure 1), we made our antigen collection by pooling protein extracts from intact and pathological human spinal cord samples to include both normal proteins as well as potential neoepitopes arising after damage. Indeed, the recognition of protein neoepitopes induced by cell damage or stress is a major characteristic of natural AAbs (26, 27) and it has been shown that two neoepitopes arising after SCI are targeted by IgM AAbs naturally present in intact mice (46). Accordingly, our results show that most AAb increased after SCI are directed against isoforms with isoelectric points and molecular weights suggestive of degraded fragments or post-translational modifications. In this regard, it should be considered that due to the lack of non-fixed human SCI tissue, the antigen collection is derived from control spinal cord samples as well as samples from patients with multiple sclerosis, Balo concentric lesions and lateral amyotrophic sclerosis. As explained before, these pathological samples were selected to include proteins that may be expressed “*de novo*” or modified by inflammation and neurodegeneration, events occurring also after traumatic SCI. We do not know whether exactly the same protein modifications are present in the spinal cord after traumatic injury, but the increase of AAbs against these modifications in SCI patients suggests that this might be the case.

Further supporting that AAbs increased after SCI are pre-existing AAbs is their rapid augmentation after injury, suggesting that they are part of secondary rather than primary humoral responses. In this regard, AAbs rise before new primary IgG responses may be detected (47), at the very acute phase in patients (Figure 4) and bound to their antigens in the spinal cord of rats at 1 day after lesion (Figure 5). Also supporting that this increase is due to secondary responses is the observation that it is independent of the neurological level of lesion, contrary to what occurs with primary humoral responses (17). Overall, our results do not distinguish whether the increased binding of IgG AAbs induced by SCI is just the result of increasing the levels of pre-existent natural AAbs or the result of maturating their affinity, but our data strongly support that increased AAbs at the subacute phase of SCI have their origin in naturally occurring AAbs.

The existence of naturally occurring IgG AAb in healthy subjects was reported in the early days of immunology and it has been a subject of research that challenges the classical view of the role of the immune system as merely defensive (44, 48, 49). Natural AAbs are originally present as germ line-encoded IgMs in newborns and as T-cell-dependent IgGs in adults (20, 22–24). Naturally occurring AAbs have been suggested to participate in physiological responses to tissue damage, such as cleaning of cellular debris, and their deficiency have been related to disease (26, 50, 51). Indeed, intravenous IgG (IVIg) preparations, which are used to treat both immunedepression and autoimmune disorders, are manufactured by pooling the serum IgGs from more than 1,000 healthy donors and are loaded with AAbs against a wide range of antigens (52). In this regard, a commercial human IVIg preparation containing IgG AAbs that bind to astrocytes, oligodendrocytes, microglia and neurons is therapeutic in experimental SCI (53), suggesting that endogenous naturally occurring AAbs might have a protective role immediately after SCI as well. A strong parallelism has been reported to occur in stroke, where pre-existing anti-NMDAR1 autoantibodies normally present in healthy subjects have an acute protective role if the blood brain barrier (BBB) is competent before occurrence of the ischemia (54, 55). However, when the BBB is chronically compromised before the insult, anti-NMDAR1 autoantibodies associates with larger lesion volumes. The access of pre-existing AAbs to the central nervous system after breakdown of the BBB has been also related with the pathogenesis of other neurological conditions (56). Unsolved issues worth of further research are whether the AAbs upregulated after SCI might also behave as a two-edged sword depending on the patient previous BBB estate and whether, additionally, these AAbs could convert into pathogenic due to their expansion, affinity maturation or other processes.

It should be also considered that although we have detected by immunohistochemistry, Western blot and 2D-Western blot the presence of these autoantibodies in the sera from sixteen intact subjects, their levels may vary depending on the age and gender of individuals (20). Further studies including a higher number of control subjects should be performed to assess the potential effect of these variables on the presence of naturally occurring autoantibodies throughout the population of healthy individuals.

### Potential implications of the augmentation of natural autoantibodies after SCI

Regardless of whether the increased AAbs detected at this time are beneficial, pathogenic and/or could be used just as biomarkers of underlying processes, we performed a gene ontology based functional enrichment analysis to infer which cellular or physiological processes associated with the AAb targets could be more likely to be affected. This resulted in a significant enrichment of functions related to cytoskeleton organization, energetic metabolism, albumin-related functions, viral latency, transport of oxygen and bone remodeling as well as glutamate biosynthesis. Regarding the first set of these processes, cytoskeleton organization, it is well-known that SCI induces changes that affect glial and neuronal cytoskeleton, such as astrocytosis, axon dystrophy and myelin disorganization (clearly observable in Figures 2, 3). Thus, the increase in anti-GFAP, anti-NFL and anti-NFM AAb levels could be contributing to cytoskeleton changes and/or reflecting that their targets are being modified. Of note, although anti-GFAP, anti-NFL and anti-NFM AAbs target intracellular proteins, it should not be discarded that AAbs could reach them *in vivo* (57, 58).

Patients also present an altered metabolic rate and several mechanisms have been proposed to explain it (59). The increase in anti-G3P, anti-ENOA, anti-KPYM, anti-PGK1, and anti-ATPA AAbs suggest that their target antigens are being modified after SCI –which could offer new perspectives on why metabolic alterations occur– and/or that metabolic alterations might be induced by an autoimmune response. In the same line of evidence, SCI patients develop osteoporosis and one of the gene ontology terms significantly enriched is “bone resorption,” associated to CAH2.

Likewise, the levels of anti-PPIA AAbs increase. This protein, also known as cyclophilin A, is the target of the immunosupressor cyclosporine A (60), which opens the question of whether anti-PPIA AAbs might be endogenously mimicking cyclosporine. Functional enrichment analysis suggests that PPIA is, in addition, related to viral responses. In this regard, PPIA is necessary for the replication and reactivation of several virus, including human Cytomegalovirus (CMV) (61), proposed to be a general cause of immunosenescence (62, 63) and more specifically of immunosenescence after SCI (64). Whether anti-PPIA AAb levels could be an underlying mechanism of immunosenescence/immunodepression after SCI is worth of further studies.

It has been previously reported that SCI-induced immune deficiency syndrome (SCI-IDS) is rapidly established after lesion (65, 66) and is dependent on the lesion level and severity (10, 15). In an apparent contradiction, our results show that the opposite phenomenon, autoimmunity, is established at the same time and is independent of lesion level and severity. Thus, if both immunedepression and autoimmunity co-exist, they are likely to be independent phenomena. Supporting this, deficient humoral reactions are circumscribed to primary responses (responses against new antigens), while secondary antibody responses (already established responses) are preserved and thus not affected by the lesion level (17). Accordingly, as previously discussed, our results suggest that at least part of the AAb responses that are induced after SCI are secondary since (i) AAbs that augment after SCI are already present in healthy subjects at lower levels, (ii) all IgG AAbs that increase at 1 month after injury are also augmented before new IgG responses may occur, at 2 days after injury, and (iii) the level and severity of injury do not affect the levels of AAbs after SCI. Therefore, the AAb response at the subacute phase might be an independent phenomenon of immune depression because is the consequence of secondary immune responses of pre-existing AAbs.

## Conclusions

Overall, our results show that a set of naturally occurring AAbs are expanded after SCI, which in the light of previous studies could help to explain why antibody-mediated autoimmunity initiates after SCI. Also, we report 16 antigenic targets that are involved in alterations that are known to occur after SCI, such as cytoskeleton remodeling, osteoporosis or metabolic rate change. Further studies are needed to elucidate the role of each AAb independently and the dynamics of their serum levels.

## Dataset availability

The raw data supporting the conclusion of this manuscript will be made available upon request, without undue reservation, to any qualified researcher.

## Author contributions

AA-M conceived and designed the study. AA-M, LG, DG-O, OM, DM, and AE further refined the study. AA-M, LG, DG-O, and OM coordinated the study. LG, EV, MA, MAA, SC, RC, FT, RP, NS-B, DM, and AE are the treating physicians of the patients included in this study and together with OM collected samples and clinical data. CR coordinated the recruitment of healthy subjects and collected control samples. AA-M, DG-O, BP-T, GB-G, AA, and AT conducted and interpreted 2D-WB studies. GB and AA conducted and interpreted mass spectrometry studies. AA-M, DG-O, and BP conducted and interpreted immunohistochemistry studies. AA-M analyzed the data. AA-M, LG, DG-O, and EM-H interpreted the data. AA-M wrote the final manuscript with assistance from LG, DG-O, GB-G, and EM-H. All authors approved the final manuscript.

### Conflict of interest statement

The authors declare that the research was conducted in the absence of any commercial or financial relationships that could be construed as a potential conflict of interest.
