# Supplementary material for: Elevated Autoantibodies in Subacute Human Spinal Cord Injury Are Naturally Occurring Antibodies
Source: Front Immunol. 2018 Oct 11;9:2365. doi: 10.3389/fimmu.2018.02365 (PMC6193075; doi:10.3389/fimmu.2018.02365)
Supplement: Supplementary file 1 [file Data_Sheet_1.PDF]

Supplementary Figure 1

ELEVATED AUTOANTIBODIES IN SUBACUTE HUMAN SPINAL CORD INJURY ARE NATURALLY OCCURRING ANTIBODIES

Angel Arevalo-Martin*, Lukas Grassner, Daniel Garcia-Ovejero, Beatriz Paniagua-Torija, Gemma Barroso-Garcia, Alba Gonzalez-Arandilla, Orpheus Mach, Angela Turrero, Eduardo Vargas, Monica Alcobendas, Carmen Rosell, Maria A. Alcaraz, Silvia Ceruelo, Rosa Casado, Francisco Talavera, Ramiro Palazón, Nuria Sanchez-Blanco, Doris Maier, Ana Esclarin, Eduardo Molina-Holgado.

* Correspondence: aarevalom@sescam.jccm.es

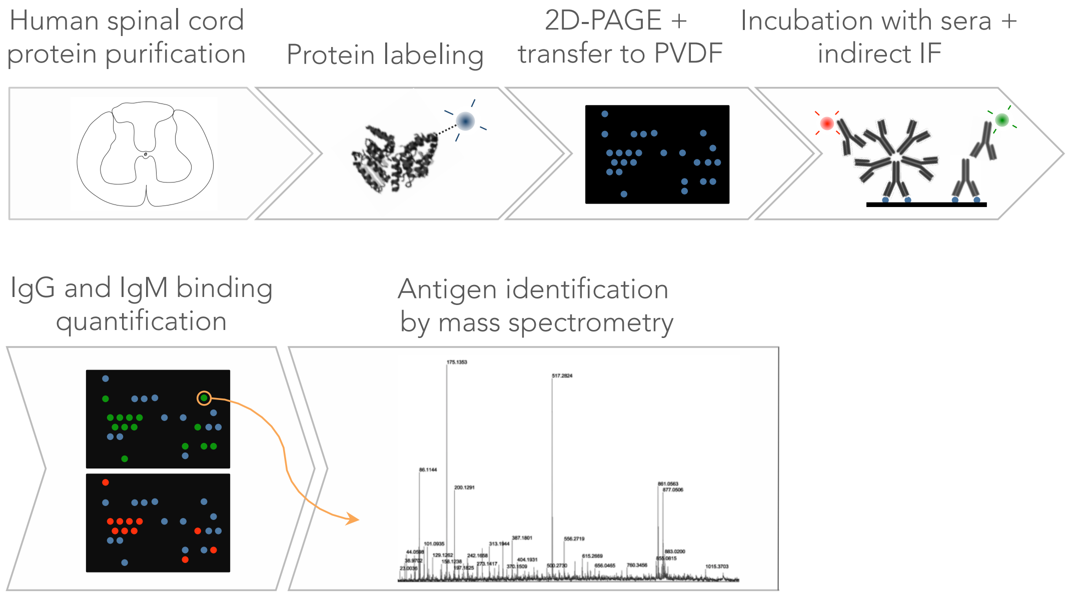

Supplementary Figure 1: Graphical overview of proteomics technics and procedures used to identify and quantify AAb from sera samples. As described in detailed in materials and methods section, proteins were extracted from human spinal cord samples from control subjects as well as from multiple sclerosis, Balo concentric lesions and amyotrophic lateral sclerosis. These proteins were pooled, fluorescently labelled and separated by two-dimensional electrophoresis. Proteins were transferred to low fluorescence PVDF membranes and were exposed to sera from control subjects and from patients. Fluorescently labelled secondary anti-human IgG and IgM antibodies were used to detect bound sera IgGs and IgMs (respectively). Binding of these sera IgG and IgM AAbs was quantified and statistically analyzed as detailed in material and methods section, while their antigenic targets were identified by mass spectrometry.
