# Supplementary material for: Elevated Autoantibodies in Subacute Human Spinal Cord Injury Are Naturally Occurring Antibodies
Source: Front Immunol. 2018 Oct 11;9:2365. doi: 10.3389/fimmu.2018.02365 (PMC6193075; doi:10.3389/fimmu.2018.02365)
Supplement: Supplementary file 2 [file Data_Sheet_2.PDF]

Supplementary Figure 2

ELEVATED AUTOANTIBODIES IN SUBACUTE HUMAN SPINAL CORD INJURY ARE NATURALLY OCCURRING ANTIBODIES

Angel Arevalo-Martin*, Lukas Grassner, Daniel Garcia-Ovejero, Beatriz Paniagua-Torija, Gemma Barroso-Garcia, Alba Gonzalez-Arandilla, Orpheus Mach, Angela Turrero, Eduardo Vargas, Monica Alcobendas, Carmen Rosell, Maria A. Alcaraz, Silvia Ceruelo, Rosa Casado, Francisco Talavera, Ramiro Palazón, Nuria Sanchez-Blanco, Doris Maier, Ana Esclarin, Eduardo Molina-Holgado.

* Correspondence: aarevalom@sescam.jccm.es

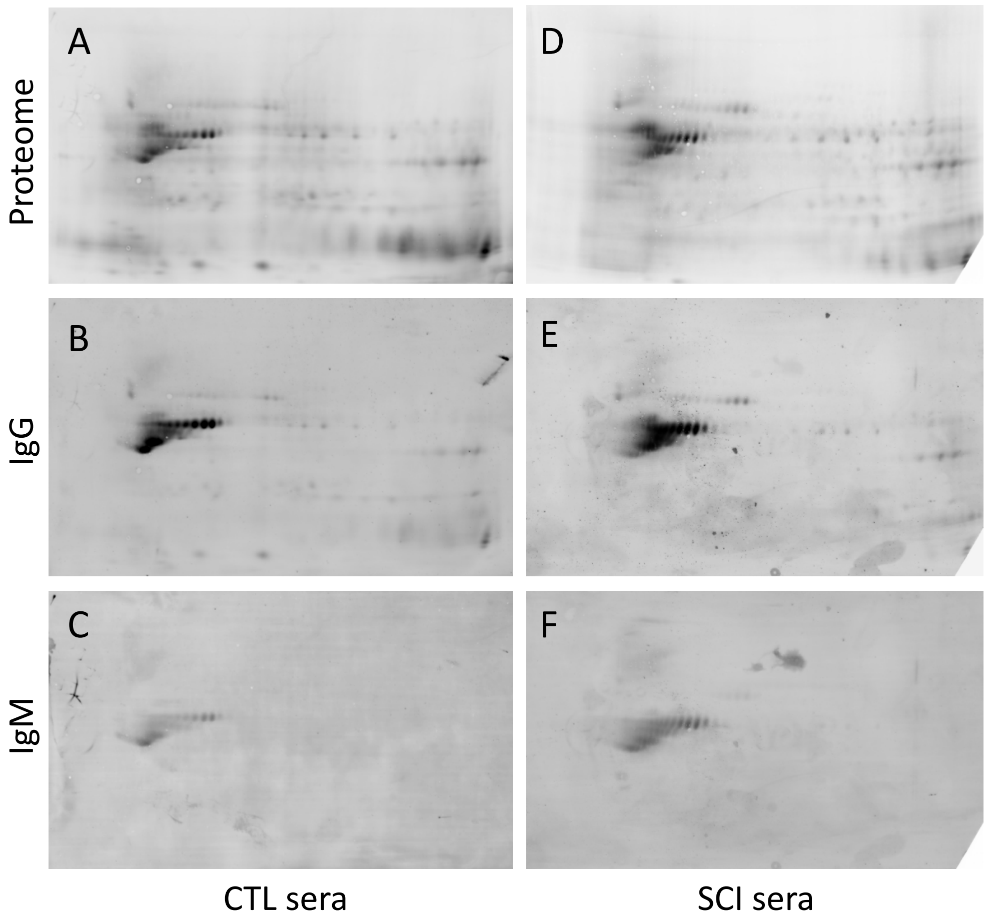

Suppl. Fig. 2: Detection of autoantibodies by two dimensional-Western blot. Gray scale converted images of Cy2-labelled spinal cord proteins after two-dimensional electrophoresis (A, D). After blotting these membranes with sera from a healthy subject (A, B, C) or from a spinal cord injury patient (D, F, G), both bound IgG (B, E) and bound IgMs (C, F) are detected. Sera IgG and IgM were detected by specific Cy3-conjugated anti-human IgG or Alexa647-conjugated anti-human IgM
