# Supplementary material for: Elevated Autoantibodies in Subacute Human Spinal Cord Injury Are Naturally Occurring Antibodies
Source: Front Immunol. 2018 Oct 11;9:2365. doi: 10.3389/fimmu.2018.02365 (PMC6193075; doi:10.3389/fimmu.2018.02365)
Supplement: Supplementary file 3 [file Data_Sheet_3.PDF]

Supplementary Figure 3

ELEVATED AUTOANTIBODIES IN SUBACUTE HUMAN SPINAL CORD INJURY ARE NATURALLY OCCURRING ANTIBODIES

Angel Arevalo-Martin*, Lukas Grassner, Daniel Garcia-Ovejero, Beatriz Paniagua-Torija, Gemma Barroso-Garcia, Alba Gonzalez-Arandilla, Orpheus Mach, Angela Turrero, Eduardo Vargas, Monica Alcobendas, Carmen Rosell, Maria A. Alcaraz, Silvia Ceruelo, Rosa Casado, Francisco Talavera, Ramiro Palazón, Nuria Sanchez-Blanco, Doris Maier, Ana Esclarin, Eduardo Molina-Holgado.

* Correspondence: aarevalom@sescam.jccm.es

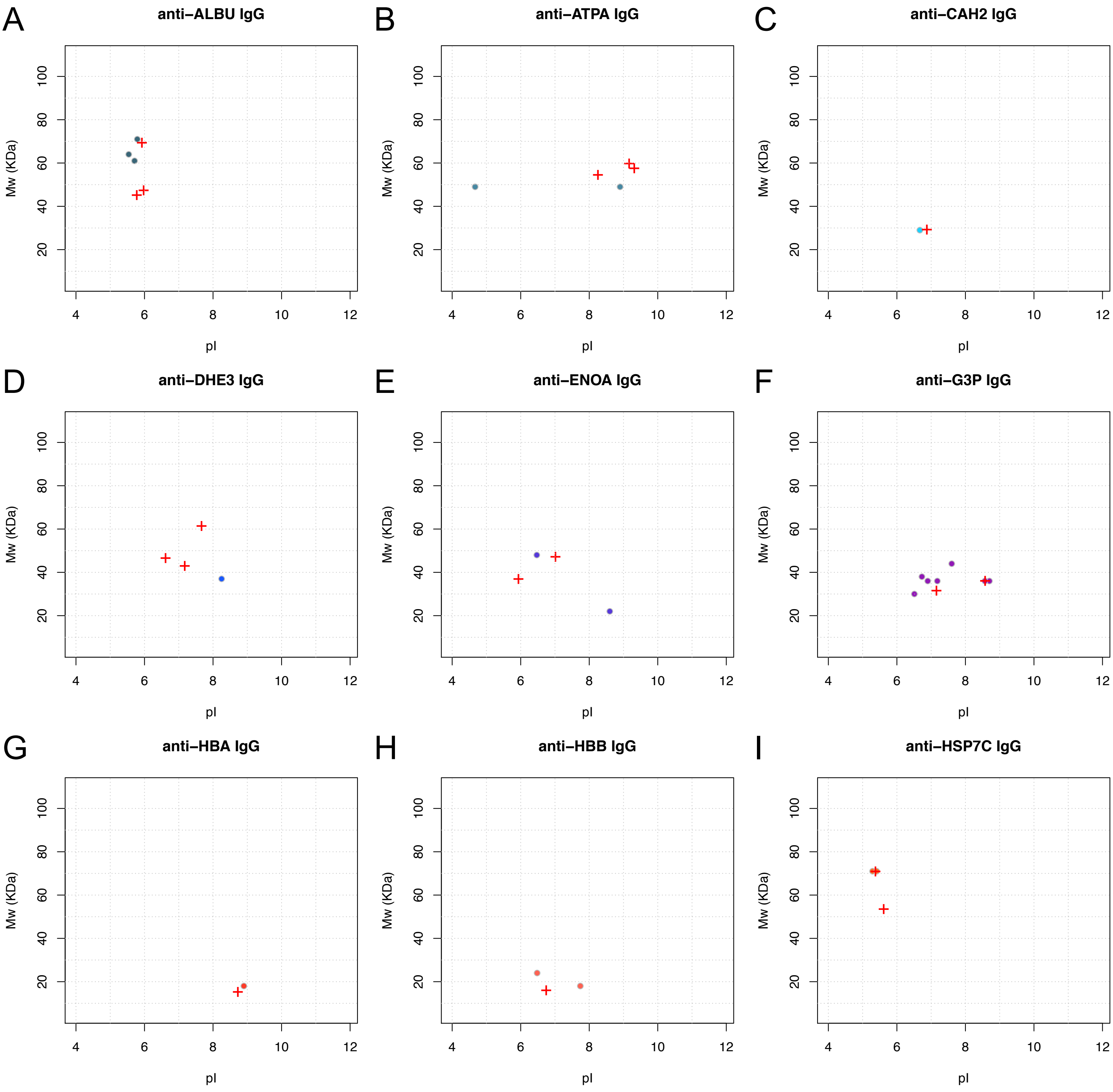

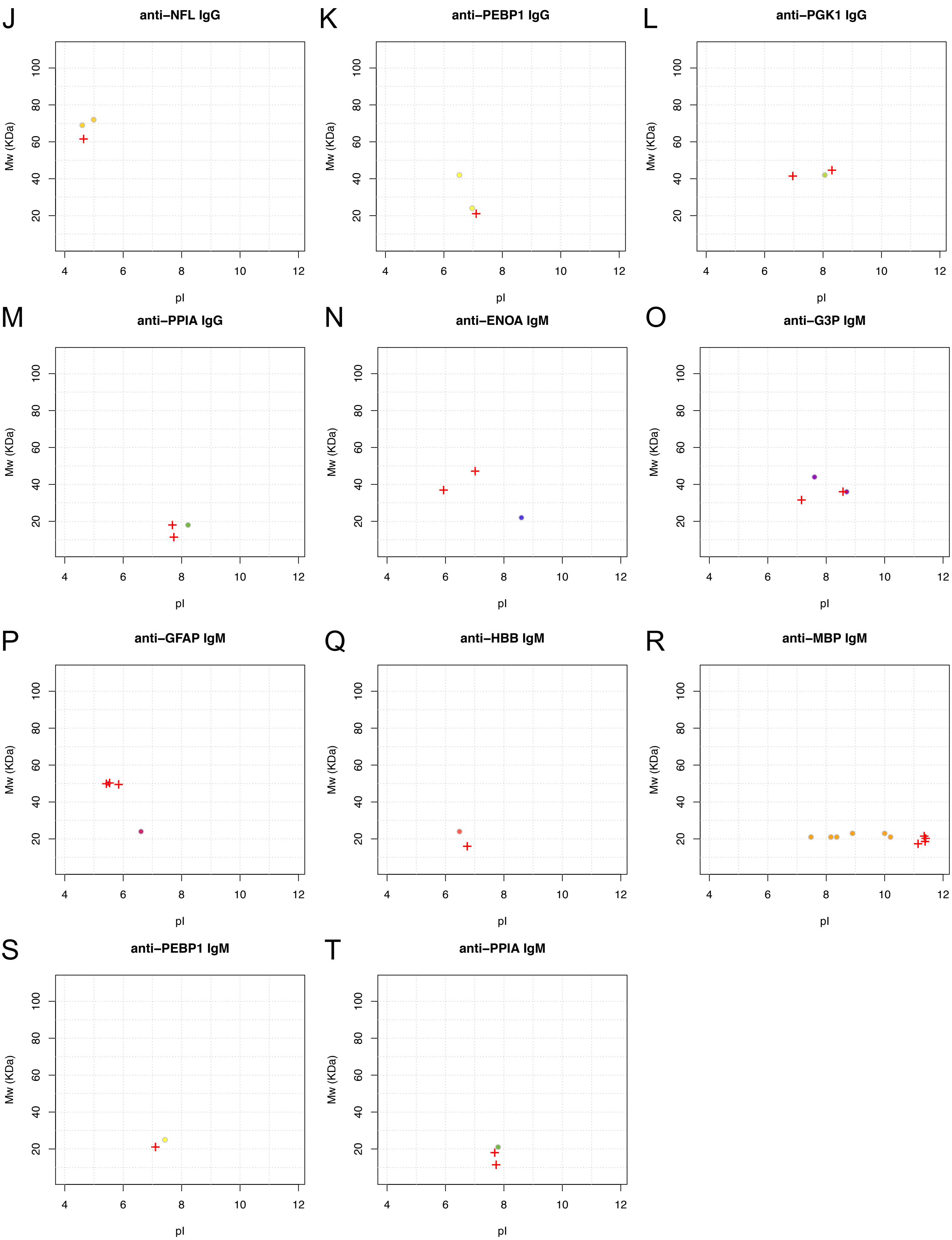
J
anti–NFL IgG
K
anti–PEBP1 IgG
L
anti–PGK1 IgG
M
anti–PPIA IgG
N
anti–ENOA IgM
O
anti–G3P IgM
P
anti–GFAP IgM
Q
anti–HBB IgM
R
anti–MBP IgM
S
anti–PEBP1 IgM
T
anti–PPIA IgM
Mw (KDa)
20 40 60 80 100
pI
4 6 8 10 12

Suppl. Fig. 3: Maps of protein isoforms targeted by autoantibodies that are increased after spinal cord injury reveals that most AAb targets present modifications that affect to their molecular weight and/or isoelectric point. (A-T) Circles represent the isoforms for which AAb are significantly increased after spinal cord injury. Red crosses mark the theoretical location of the basal isoform of a given protein. When multiple red crosses are shown for a protein, each correspond to a known alternative splicing isoform. A general displacement of AAb targets with respect to predicted basal isoforms of each protein is observed, both on molecular weight and isoelectric point.
