# Supplementary material for: Elevated Autoantibodies in Subacute Human Spinal Cord Injury Are Naturally Occurring Antibodies
Source: Front Immunol. 2018 Oct 11;9:2365. doi: 10.3389/fimmu.2018.02365 (PMC6193075; doi:10.3389/fimmu.2018.02365)
Supplement: Supplementary file 4 [file Data_Sheet_4.PDF]

Supplementary Table 1

ELEVATED AUTOANTIBODIES IN SUBACUTE HUMAN SPINAL CORD INJURY ARE NATURALLY OCCURRING ANTIBODIES

Angel Arevalo-Martin*, Lukas Grassner, Daniel Garcia-Ovejero, Beatriz Paniagua-Torija, Gemma Barroso-Garcia, Alba Gonzalez-Arandilla, Orpheus Mach, Angela Turrero, Eduardo Vargas, Monica Alcobendas, Carmen Rosell, Maria A. Alcaraz, Silvia Ceruelo, Rosa Casado, Francisco Talavera, Ramiro Palazón, Nuria Sanchez-Blanco, Doris Maier, Ana Esclarin, Eduardo Molina-Holgado.

* Correspondence: aarevalom@sescam.jccm.es

Suppl. Table 1: Characteristics of spinal cord tissue samples employed for proteomics studies

Code	Diagnostic[a]	Gender	Age	Spinal cord level	PMD[b] (h:m)
BCPA364M	No SC pathology	M	43	cervical	Unknown
BCPA188M	No SC pathology	M	46	cervical	Unknown
BCPA236M	No SC pathology	F	52	cervical	Unknown
CS1113	No SC pathology	M	85	thoracic	8:15
BCPA295M	ALS	F	59	thoracic	Unknown
BCPA325M	ALS	F	56	thoracic	Unknown
CS1036	ALS	M	38	thoracic	7:00
CS946	ALS + ARP III	F	84	lumbar	5:00
CS840	MS	M	68	thoracic	7:00
CS921	MS	F	52	lumbar	3:30
CS1598	Balo concentric sclerosis	M	32	cervical/ thoracic	10:00

[a] SC: spinal cord; ALS: amyotrohic lateral sclerosis; ARP III: Alzheimer's disease related pathology, Braak stage III; MS: multiple sclerosis.
[b] PMD: Postmortem delay (in hours:minutes)
