# Supplementary material for: Elevated Autoantibodies in Subacute Human Spinal Cord Injury Are Naturally Occurring Antibodies
Source: Front Immunol. 2018 Oct 11;9:2365. doi: 10.3389/fimmu.2018.02365 (PMC6193075; doi:10.3389/fimmu.2018.02365)
Supplement: Supplementary file 5 [file Data_Sheet_5.PDF]

Supplementary Table 3

ELEVATED AUTOANTIBODIES IN SUBACUTE HUMAN SPINAL CORD INJURY ARE NATURALLY OCCURRING ANTIBODIES

Angel Arevalo-Martin*, Lukas Grassner, Daniel Garcia-Ovejero, Beatriz Paniagua-Torija, Gemma Barroso-Garcia, Alba G Arandilla, Orpheus Mach, Angela Turrero, Eduardo Vargas, Monica Alcobendas, Carmen Rosell, Maria A. Alcaraz, Silvia Ceruelo, Rosa Casado, Francisco Talavera, Ramiro Palazón, Nuria Sanchez-Blanco, Doris Maier, Ana Esclarin, Eduardo Molina-Holgado.

* Correspondence: aarevalom@sescam.jccm.es

Suppl. Table 3: Hematological values of control subjects, central cord syndrome patients and cervical AIS A patients

Group[a]	Sample size	Days after injury	Erythrocytes (10^9/ml)	Platelets (10^6/ml)	Leukocytes (10^6/ml)	Neutrophils (10^6/ml)	Lymphocytes (10^6/ml)	Monocytes (10^6/ml)	Eosinophils (10^6/ml)	Basophils (10^6/ml)
CTL	16		5.0±0.1	243±12.4	6.3±0.3	3.7±0.2	1.9±0.1	0.5±0.04	0.1±0.01	0.006±0.006
CCS	10	27.6±2.3	4.4±0.1 **	297±21.9 *	6.4±0.6	3.9±0.5	1.7±0.1	0.5±0.06	0.2±0.03	0.02±0.012
Cerv AIS A	14	30.5±2.0	3.8±0.1 *** ††	373±39.7 **	8.3±0.7 *	4.9±0.6 *	1.2±0.1 **†	0.5±0.06	0.2±0.08	0.01±0.010

[a] CTL: control healthy subjects. CCS: traumatic central cord syndrome patients. Cerv AIS A: cervical traumatic AIS A patients.

* t-test vs CTL p-value<0.05; ** t-test vs CTL p-value<0.01; *** t-test vs CTL p-value<0.001; † t-test vs CCS p-value<0.05; †† t-test vs CCS p-value<0.01.
